# A conductive catecholate-based framework coordinated with unsaturated bismuth boosts CO_2_ electroreduction to formate†

**DOI:** 10.1039/d3sc01876h

**Published:** 2023-05-31

**Authors:** Zengqiang Gao, Man Hou, Yongxia Shi, Li Li, Qisheng Sun, Shuyuan Yang, Zhiqiang Jiang, Wenjuan Yang, Zhicheng Zhang, Wenping Hu

**Affiliations:** a Department of Chemistry, School of Science, Tianjin Key Laboratory of Molecular Optoelectronic Sciences, Tianjin University Tianjin 300072 China zczhang19@tju.edu.cn huwp@tju.edu.cn; b Julong College, Shenzhen Technology University Shenzhen 518118 China wj914315@163.com; c Haihe Laboratory of Sustainable Chemical Transformations Tianjin 300192 China; d Joint School of National University of Singapore and Tianjin University, International Campus of Tianjin University Binhai New City Fuzhou 350207 China; e Vanadium and Titanium Resource Comprehensive Utilization Key Laboratory of Sichuan Province, Panzhihua University Panzhihua Sichuan 617000 P. R. China

## Abstract

Bismuth-based metal–organic frameworks (Bi-MOFs) have received attention in electrochemical CO_2_-to-formate conversion. However, the low conductivity and saturated coordination of Bi-MOFs usually lead to poor performance, which severely limits their widespread application. Herein, a conductive catecholate-based framework with Bi-enriched sites (HHTP, 2,3,6,7,10,11-hexahydroxytriphenylene) is constructed and the zigzagging corrugated topology of Bi–HHTP is first unraveled *via* single-crystal X-ray diffraction. Bi–HHTP possesses excellent electrical conductivity (1.65 S m^−1^) and unsaturated coordination Bi sites are confirmed by electron paramagnetic resonance spectroscopy. Bi–HHTP exhibited an outstanding performance for selective formate production of 95% with a maximum turnover frequency of 576 h^−1^ in a flow cell, which surpassed most of the previously reported Bi-MOFs. Significantly, the structure of Bi–HHTP could be well maintained after catalysis. *In situ* attenuated total reflectance Fourier transform infrared spectroscopy (ATR-FTIR) confirms that the key intermediate is *COOH species. Density functional theory (DFT) calculations reveal that the rate-determining step is *COOH species generation, which is consistent with the *in situ* ATR-FTIR results. DFT calculations confirmed that the unsaturated coordination Bi sites acted as active sites for electrochemical CO_2_-to-formate conversion. This work provides new insights into the rational design of conductive, stable, and active Bi-MOFs to improve their performance towards electrochemical CO_2_ reduction.

## Abstract 2

A conductive MOF, named Bi-based catecholate BiHHTP, exhibited excellent catalytic performance towards CO_2_ electroreduction.

## Graphical abstract

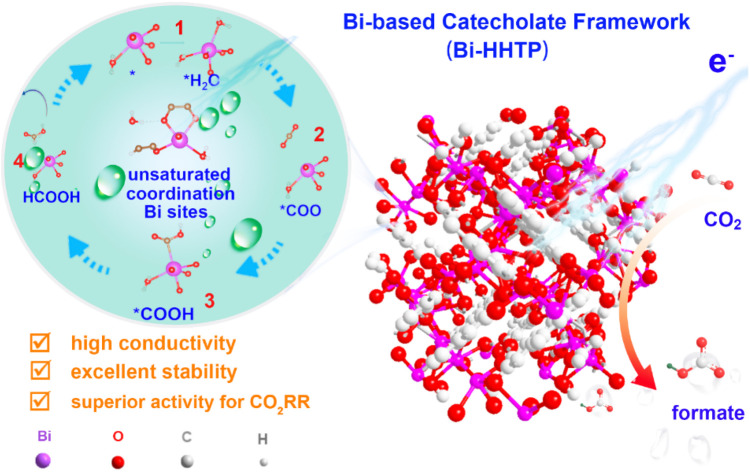

## Introduction

With ever-increasing global energy demand and the influence of climate change, the electrochemical CO_2_ reduction reaction (CO_2_RR) has attracted enormous attention for converting CO_2_ into value-added fuels and feedstocks, which is not only a promising way to reduce the negative impact of climate change but also enriches energy supply and achieves net-zero CO_2_ emission.^1,2^ Formate, as one of the main CO_2_RR products, has promising potential because of its compatibility with existing infrastructure and the promise for hydrogen storage and textile or leather production, proposing a carbon-neutral route to fuel generation.^3–6^

Electrocatalysts play a vital role in efficient CO_2_-to-formate conversion.^4,7,8^ Bismuth (Bi) with its low-cost and environment-friendly nature exhibits adequate adsorption intensity toward intermediate species for formate, attracting more and more attention.^9–11^ Considering its low natural abundance and potential future large-scale applications, it is crucial to enhance the atomic utilization of Bi. Bi-based metal–organic frameworks (Bi-MOFs) with well-defined coordination and single-site dispersion are regarded as promising approaches to address this issue.^12,13^ But their low electrical conductivity and saturated coordination mode remain significant obstacles to their performance and severely limit their broad application. Spectacularly, conductive MOFs (cMOFs) are deemed proactive materials for achieving excellent performance without the pyrolysis process owing to their distinctive electrical conductivity.^14–21^ Despite the advances in the CO_2_RR with cMOFs such as Cu-based cMOFs,^22–24^ there is still a scarcity of literature on conductive Bi-MOFs for electrochemical CO_2_-to-formate conversion with enhanced activity and satisfactory selectivity.

In this work, a three-dimensional (3D) Bi-based catecholate cMOF (Bi–HHTP) (HHTP, 2,3,6,7,10,11-hexahydroxytriphenylene) with enriched defects was fabricated for the CO_2_RR and single-crystal X-ray diffraction verified its structure. Bi–HHTP with its robust nature could achieve optimal faradaic efficiency (FE) towards formate (95%) with a maximum turnover frequency (TOF) of ∼576 h^−1^. *In situ* attenuated total reflectance Fourier transform infrared spectroscopy (ATR-FTIR) and density functional theory (DFT) calculations confirmed that the key intermediate is *COOH species. DFT calculations indicated that the unsaturated coordination Bi^3+^ sites could effectively facilitate the dissociation of *COOH to produce formate.

## Results and discussion

### Preparation and characterization

**Fig. 1:**
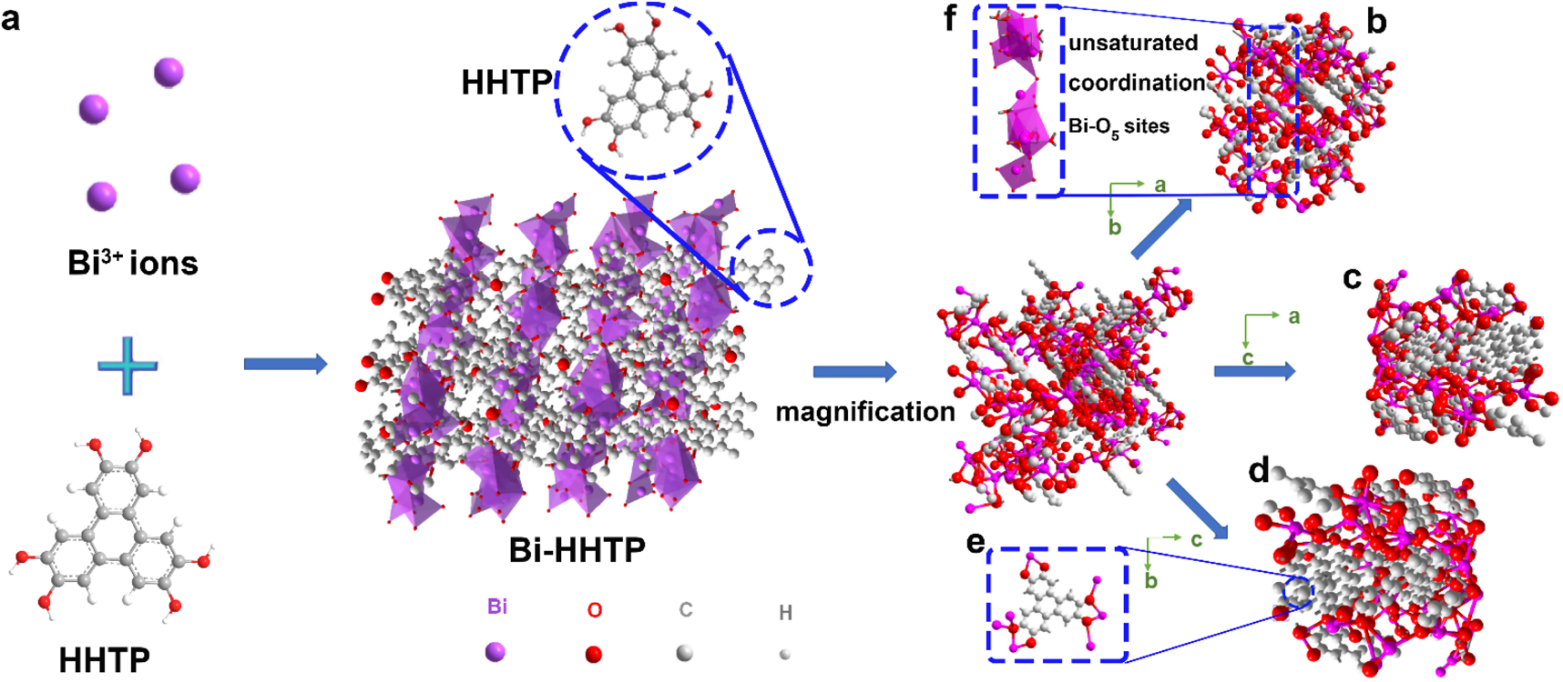
(a) The general process for the synthesis of Bi–HHTP. (b–d) The views in a different dimension. (e) Chelation of HHTP toward Bi^3+^ ions. (f) The coordination environment of Bi^3+^ ions (distorted tetragonal pyramid).

**Fig. 2:**
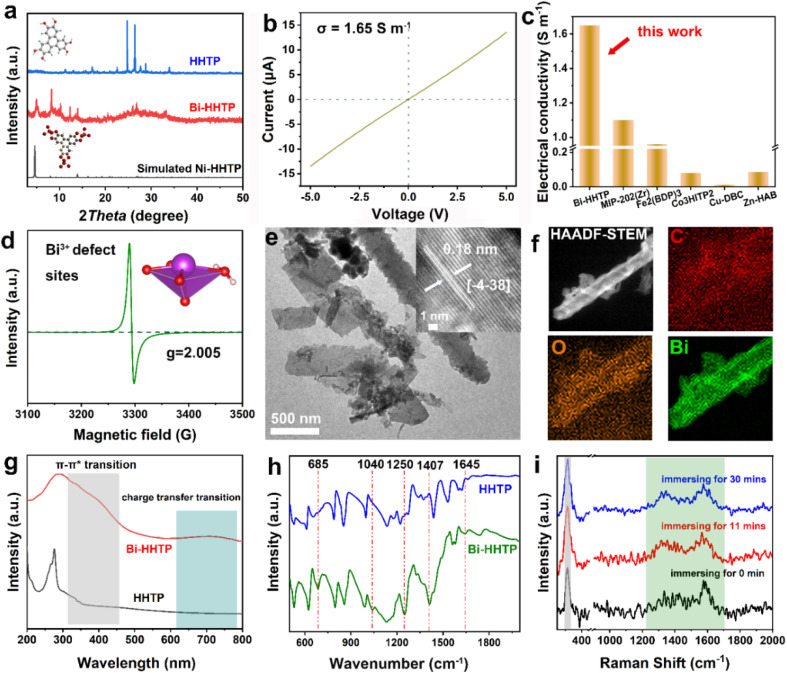
(a) XRD patterns of Bi–HHTP and HHTP. (b) Current–voltage characteristic of Bi–HHTP using the two-contact probe method. (c) The compassion of conductivity of different MOFs. (d) EPR spectrum of Bi–HHTP. (e) TEM image, (f) HAADF-STEM image and the corresponding EDS elemental mappings of Bi–HHTP. (g) UV-vis spectra of Bi–HHTP and HHTP. (h) FTIR and (i) *in situ* Raman spectra of Bi–HHTP.

In this work, 3D Bi–HHTP was constructed *via* the solvothermal method (Fig. 1). Single-crystal X-ray diffraction (XRD) disclosed that Bi–HHTP exhibited a monoclinic type Bravais lattice (*α*, *γ* = 90° and *β* = 143.57(2)°) with space group *P*2_1_/*n*. Intriguingly, the zigzagging corrugated chains of nonplanar ligands were coupled by the Bi–O bond (Fig. 1a–d), where each HHTP attached to seven Bi^3+^ ions at different angles (Fig. 1e) and the Bi^3+^ ion coordinated with the nonplanar catechol groups to form the unsaturated coordination mode (distorted tetragonal pyramid) (Fig. 1f). XRD patterns in Fig. 2a further confirmed the difference in Bi–HHTP, compared to traditional Ni–HHTP. No preferential orientation was found in Bi–HHTP. Thereafter, the conductivity of Bi–HHTP was measured by the two-contact probe technique. According to the current–voltage characteristic shown in Fig. 2b, Bi–HHTP exhibited an electrical conductivity of 1.65 S m^−1^, which is higher than those of the reported MOFs as listed in Fig. 2c and Table S2.† Electron paramagnetic resonance spectroscopy (EPR) could effectively disclose the existence of unpaired electrons.^25^ The EPR spectrum of Bi–HHTP (Fig. 2d) revealed a high-intensity peak at *g* = 2.005, verifying the presence of unpaired electrons located on defect sites. Transmission electron microscopy (TEM) and scanning electron microscopy (SEM) images showed that the morphology of Bi–HHTP was nanobelt-like (Fig. 2e, S1a and b†). The lattice fringes of Bi–HHTP in specific domains with lattice parameters of 0.18 nm matched well with [−4 −3 8] of Bi–HHTP (inset of Fig. 2e). The composition of Bi–HHTP was analyzed using the high-angle annular dark-field scanning transmission electron microscopy (HAADF-STEM) image and the corresponding energy-dispersive X-ray spectroscopy (EDS) elemental mappings (Fig. 2f), indicating the homogeneous distribution of Bi, C, and O elements.

Besides, UV-vis and FTIR were also employed to detect the feature of Bi–HHTP. The peaks of UV-vis spectra shown in Fig. 2g at ∼380 nm and ∼700 nm correspond to π–π* and the ligand-to-metal charge transfer between the Bi^3+^ ion and HHTP.^26,27^ Moreover, the peak at 685 cm^−1^ for FTIR shown in Fig. 2h was attributed to the vibration of the Bi–O entities, providing convincing evidence for forming Bi–HHTP.^16,28,29^ Furthermore, we carried out *in situ* Raman tests to prove the robust stability of Bi–HHTP in 0.5 M KHCO_3_ aqueous solution (Fig. 2i). The stretching mode of Bi–O entities was found at 313 cm^−1^.^30^ The peaks at around 1335 and 1582 cm^−1^ could be attributed to the characteristic defect (D) and graphitic (G) bands.^20,26,31^ Remarkably, the unchanged Bi–O entities in KHCO_3_ aqueous solution illustrated the robust structure of Bi–HHTP, compared to the reported Bi-based MOFs.^12,19^

### Electrochemical performance

**Fig. 3:**
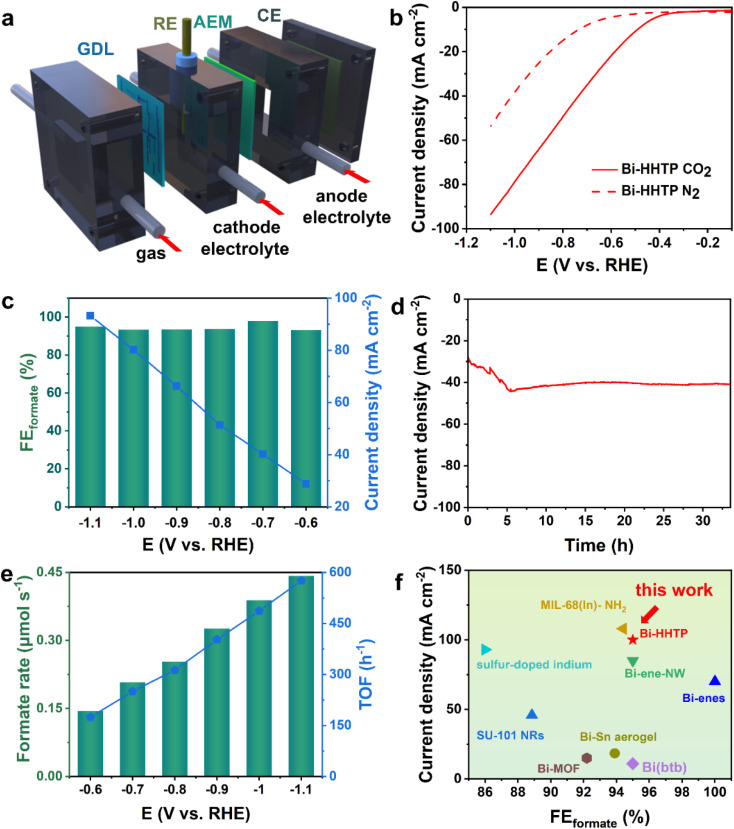
(a) The illustration scheme of a liquid-phase flow cell device. (GDL, CE, RE, and AEM represent the working electrode, counter electrode, reference electrode, and anion exchange membrane, respectively) (b) LSV curves of Bi–HHTP in CO_2_ and N_2_ atmospheres without correction. (c) Potential-dependent formate FEs and current density of Bi–HHTP. (d) Stability test of Bi–HHTP at −0.7 V *vs.* RHE. (e) Formate rate and TOF of Bi–HHTP. (f) Comparison of our work with previously reported literature.

The CO_2_RR performance was investigated in a flow cell (Fig. 3a) and the strong alkaline solution could enhance the conductivity of the electrolyte (0.194 S cm^−1^, 1 M KOH) and lower the overpotential.^32,33^ The liquid-phase products were detected by nuclear magnetic resonance (Fig. S4†). The polarization curves over Bi–HHTP in CO_2_-purged cells shown in Fig. 3b illustrated a more positive onset potential and dramatically larger current densities than those in N_2_-purged cells, signifying that Bi–HHTP could lower the reaction barriers for the CO_2_RR. Inspiringly, the FE_formate_ of Bi–HHTP shown in Fig. 3c could exceed 95% with a cathodic energy efficiency (CEE) of 68.8% at −0.7 V *vs.* RHE. The current density of Bi–HHTP was 93 mA cm^−2^ at −1.1 V *vs.* RHE.

Afterward, there was no discernible change in current density for over 30 h (Fig. 3d). The formate produced by Bi–HHTP at various potentials (Fig. 3e) illustrated that the generation rate could exceed 0.4 μmol s^−1^ and the TOF of Bi–HHTP could reach a maximum of 576 h^−1^. Meanwhile, Bi–HHTP demonstrated a high FE of formate with a high current density, which outperforms most of the reported Bi-based or In-based materials (Fig. 3f and Table S3†). To boost the electrocatalytic activity of Bi–HHTP, this work also replaced the oxygen evolution reaction (OER) with the methanol oxidation reaction (MOR) (Fig. S9–S18†) to promote reaction kinetics. Inspiringly, the pair of CO_2_RR//MOR systems only needed −1.9 V to achieve a current density of 10 mA cm^−2^, much lower (about 400 mV) than that for the CO_2_RR//OER (Fig. S19†). Moreover, the produced formate was quantitatively detected after electrolysis. Notably, the FE_formate_ for the CO_2_RR was over 90% from −2.1 to −2.5 V.

### *In situ* ATR-FTIR spectroscopy and DFT calculations

**Fig. 4:**
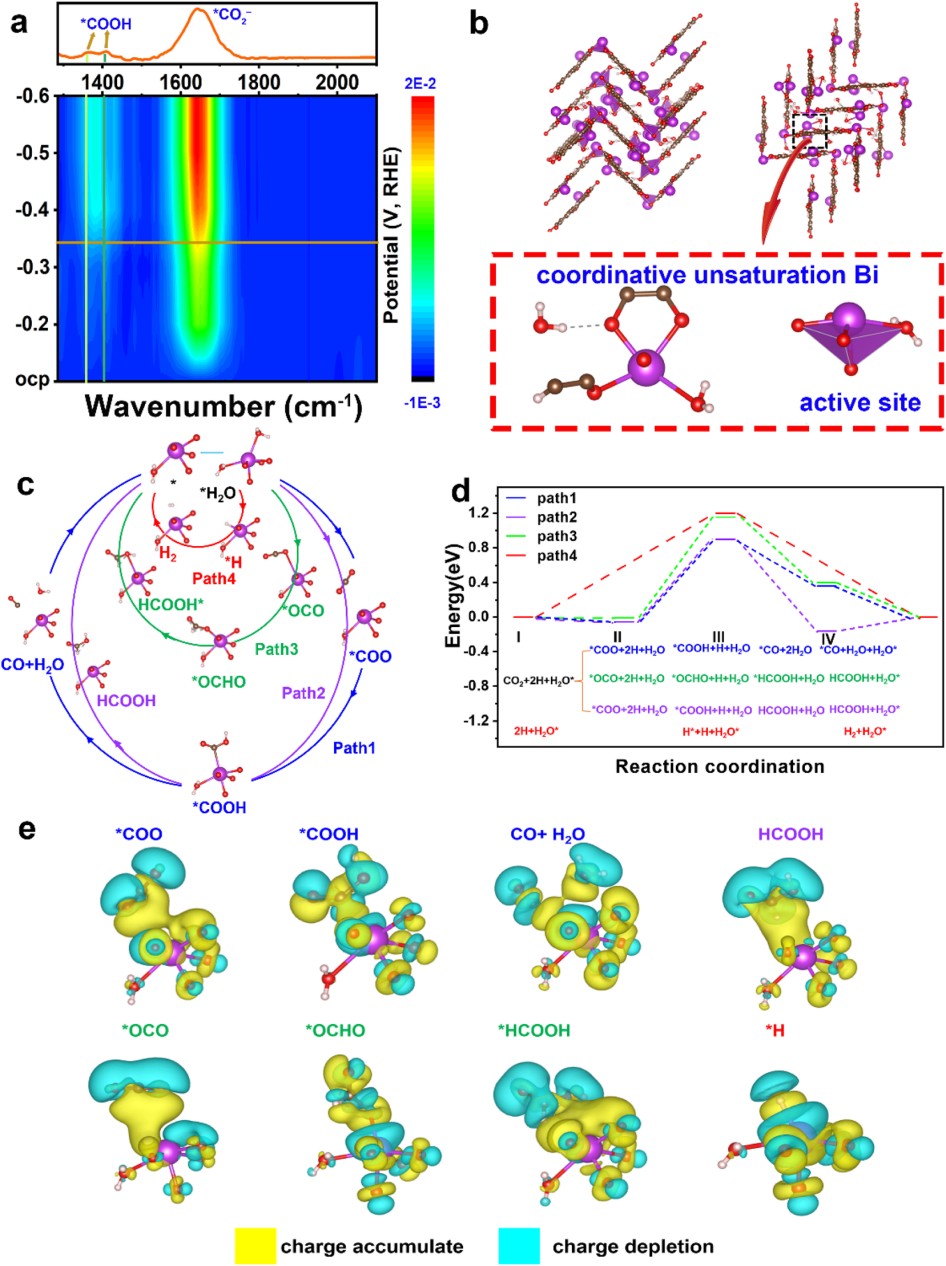
(a) *In situ* ATR-FTIR spectra in CO_2_-saturated 0.5 M KHCO_3_ at applied potentials. (b) The active site Bi coordinative unsaturation with 4 oxygen atoms and one H_2_O molecule bonded. (c) Four proposed reaction pathways on the active site to different products corresponding to the CO_2_RR and HER. (d) The energy profiles in eV for the four pathways proposed in (c) with active compositions. (e) The charge difference of every active species (*COO, *COOH, CO + H_2_O, HCOOH, *OCO, *OCHO, *HCOOH, and *H) on the catalyst surface. The yellow part indicates the charge accumulation and the cyan part illustrates the charge depletion.

To ascertain the intermediates and gain an understanding of the structure–performance relationship, *in situ* ATR-FTIR spectra were recorded and DFT calculations were applied. As shown in Fig. 4a, two FTIR signals at ∼1379 and 1410 cm^−1^ are assigned to the formation of *COOH species,^33^ and the peak at 1643 cm^−1^ arises from the vibration frequency of the carboxylate (*CO_2_^−^).^34–36^

The structural pattern of Bi–HHTP may be regarded as being composed of BiO_4_–H_2_O joined by organic linkers (HHTP). The Bi^3+^ ions have an unusual unsaturated coordination in their structure. As opposed to the saturated coordination of Bi^3+^ ions with six chemical bonds, the Bi site relates to four oxygen atoms and one water molecule (Fig. 4b). Fig. S20† illustrates the density of states of active Bi coordinative unsaturation. Four potential routes of unsaturated coordination Bi^3+^ sites for the CO_2_RR are provided (paths 1–4 in Fig. 4c). Path 1 produces CO, paths 2 and 3 result in generating HCOOH, and path 4 is the hydrogen evolution reaction (HER) pathway. The active site is denoted as H_2_O* (* denotes the adsorption state) because the active Bi^3+^ sites were distorted tetragonal pyramids and one of the ligands is H_2_O. In Fig. 4d, the energy profiles are displayed, showing that step II indicates the rate-determining step (RDS) and paths 1 and 2 are the best routes for producing HCOOH due to the lower RDS energy barrier (0.897 eV), compared to path 3 (1.15 eV) and path 4 (1.2 eV). Additionally, the blue value (path 1) is higher than the purple data (path 2). The HCOOH formation from step IV is preferable to CO generation, which is consistent with the *in situ* ATR-FTIR signals at ∼1379 and 1410 cm^−1^. The stronger selectivity for HCOOH shown between paths 1 and 2 is because *COOH on the Bi^3+^ site is strong enough to weaken the carbon–oxygen double bond. Path 4 for the HER has the highest energy barrier of 1.2 eV, which indicates that the generation of H_2_ is unfavorable.^37^ Therefore, the CO_2_RR is preferable to the HER because CO_2_ is easier to adsorb than H. The charge difference of every active species (*COO, *COOH, CO + H_2_O, HCOOH, *OCO, *OCHO, *HCOOH, and *H) on the catalyst surface is displayed in Fig. 4e. The yellow part indicates the charge accumulation and cyan part illustrates the charge depletion. Much charge transfer could be found between the unsaturated coordination Bi^3+^ sites and active species. The experimental *in situ* ATR-FTIR results and the DFT data both show that Bi–HHTP exhibits a strong selectivity for HCOOH production.

## Conclusions

In summary, this work fabricates conductive Bi–HHTP *via* the solvothermal method, and its crystal structure is first unravelled *via* single-crystal X-ray diffraction. Bi–HHTP with excellent electrical conductivity (1.65 S m^−1^) exhibits numerous defects, which is verified by EPR. Bi–HHTP displays high selectivity to formate (95%) in a flow cell with a CEE of 68.8% and a maximum TOF of 576 h^−1^. The *in situ* Raman spectroscopy, XRD, and XPS results further confirm its robust structure. Both *in situ* ATR-FTIR and DFT calculations confirm that the key intermediate is *COOH species. DFT calculations also confirm that the unsaturated coordination Bi sites within Bi–HHTP serve as active sites, which could promote charge transfer and lower the energy barrier (0.897 eV) for producing HCOOH. Besides, the electrocatalytic activity of Bi–HHTP is boosted by replacing the OER with the MOR, illustrating that the CO_2_RR//MOR system requires less overpotential (about 400 mV) than the CO_2_RR//OER to achieve a current density of 10 mA cm^−2^. This work paves a new way into constructing highly active and stable cMOFs for high-performance CO_2_RR, in terms of experimental and theoretical aspects.

## Data availability

All relevant data are presented in the main text and ESI.†

## Author contributions

Zengqiang Gao wrote the original draft and carried out the characterization and catalysis. Man Hou, Yongxia Shi, and Li Li participated in the characterization and helped draft the manuscript. Qisheng Sun and Zhiqiang Jiang joined the discussion of the data and gave useful suggestions. Shuyuan Yang participated in the characterization. Zhicheng Zhang, Wenjuan Yang and Wenping Hu supervised and guided the project.

## Conflicts of interest

There are no conflicts to declare.

## Supplementary Material

SC-014-D3SC01876H-s001

SC-014-D3SC01876H-s002
