# Supplementary material for: A conductive catecholate-based framework coordinated with unsaturated bismuth boosts CO2 electroreduction to formate
Source: Chem Sci. 2023 May 31;14(25):6860–6. doi: 10.1039/d3sc01876h (PMC10306104; doi:10.1039/d3sc01876h)
Supplement: SC-014-D3SC01876H-s001 [file SC-014-D3SC01876H-s001.pdf]

Electronic Supplementary Material (ESI) for Chemical Science.
This journal is © The Royal Society of Chemistry 2023

Conductive Catecholate-based Framework Coordinated with Unsaturated Bismuth Boosts CO_2 Electroreduction to Formate

Zengqiang Gao [a], Man Hou [a], Yongxia Shi [a], Li Li [a], Qisheng Sun [a], Shuyuan Yang [a], Zhiqiang Jiang [e], Wenjuan Yang [*b], Zhicheng Zhang [* a], Wenping Hu [* a, c, d]

[a] Department of Chemistry, School of Science; Tianjin Key Laboratory of Molecular Optoelectronic Sciences. Tianjin University. Tianjin 300072, China.

[b] Julong College, Shenzhen Technology University, Shenzhen, 518118, China.

[c] Haihe Laboratory of Sustainable Chemical Transformations, Tianjin 300192, China.

[d] Joint School of National University of Singapore and Tianjin University, International Campus of Tianjin University, Binhai New City, Fuzhou 350207, China.

[e] Vanadium and Titanium Resource Comprehensive Utilization Key Laboratory of Sichuan Province, Panzhihua University, Panzhihua, Sichuan 617000, P. R. China

*E-mails: zczhang19@tju.edu.cn; yangwenjuan@sztu.edu.cn; huwp@tju.edu.cn

Experimental Section

Reagents. Bismuth nitrate pentahydrate ($Bi(NO_3)_3 \cdot 5H_2O$), potassium hydroxide (KOH), Nafion ionomer (5 wt%), and dimethyl sulfoxide (DMSO) were purchased from Sigma-Aldrich. Nickel foam (NF), terephthalic acid, and 2,3,6,7,10,11-hexahydroxytriphenylene were purchased from TCL. Nickel acetate ($Ni(CH_3COO)_2 \cdot 4H_2O$) and Indium(III) nitrate tetrahydrate ($In(NO_3)_3 \cdot 4H_2O$) were purchased from Aladdin. The anion exchange membrane and Segracet 29BC carbon paper (CP) were purchased from Dioxide Materials. The water used in all experiments was ultrapure. All reagents were used as received without further purification.

Characterization. Scanning electron microscope (SEM) image was characterized by Hitachi SU8010 field emission SEM. Optical microscope (OM) images were characterized by Nikon ECLIPSE Ci-POL POM. X-ray diffractometer (XRD) analysis was characterized by Rigaku SmartLab XRD with monochromatic radiation of Cu Kα (λ = 1.5406 Å). Transmission electron microscopy (TEM), high-resolution transmission electron microscopy (HRTEM), high angle annular dark field scanning TEM (HAADF-STEM), and energy dispersive spectroscopy (EDS) elemental mapping investigations were performed on a TecnaiG2F20 microscope operated at 200 kV. X-ray photoelectron spectroscopy (XPS) was collected on an ESCALAB-250Xi spectrometer (Thermo Fisher Scientific) using Al Kα radiation. Electrical performances were measured by a Keithley 4200-SCS semiconductor parameter analyzer with a micromanipulator 6150 probe station. Fourier transform infrared spectroscopy (FT-IR) data were obtained by Bruker Vertex70 Fourier transform infrared spectrometer. Ultraviolet-visible diffuse reflectance (UV-vis) spectroscopy was measured by Shimadzu UV-3600Plus UV-vis spectrophotometer. The single-crystal data was collected by a XtaLAB Fr-X X-ray single-crystal diffractometer with Oxford 800 Cryostream 80 - 400 K and processed using CrysAlisPro. The structure was solved and refined using Full-matrix least-squares based on F2 with the program ShelXT and ShelXL within Olex2. More refined data are provided in Table S1.

Synthesis of Bi-HHTP. A solid mixture of HHTP (7 mg) and $Bi(NO_3)_3 \cdot 5H_2O$ (18 mg) was added to deionized water (9 mL) in a Teflon-lined autoclave. The vial was

then capped and sonicated for 30 minutes until the solid was dissolved. The reaction mixture was heated in an oven at 120°C for 12 h to produce small dark crystals. The autoclave was allowed to cool naturally, and the obtained crystals were washed with deionized water for five times.

Synthesis of In-HHTP. A solid mixture of HHTP (7 mg) and $In(NO_3)_3 \cdot 5H_2O$ (10 mg) was added to deionized water (4 mL) in a glass vial. The reaction mixture was then heated in an oven at 85°C for 12 h to produce small dark crystals. The vial was allowed to cool naturally and the obtained crystals were washed with deionized water for five times.

Big crystals of Bi-HHTP. The big crystals could be synthesized as a phase mixture under hydrothermal conditions by adding $Bi(NO_3)_3 \cdot 5H_2O$ (18 mg) and HHTP (7 mg) to a Teflon-lined autoclave containing 9 mL deionized water. The reactor was then heated to 120°C for 100 h. The products were then filtered off and washed with deionized water for three times and then dried at 60°C overnight.

Working electrode preparation for electrochemical CO_2 reduction. Catalyst (10 mg) was uniformly ground to powder and dispersed into a mixed solution of 100 μL of Nafion solution (5 wt.%) and 850 μL of isopropanol solution, then sonicated for 60 min to form a homogeneous ink. 100 μL of the well-dispersed ink was loaded onto a CP with an area of 1 cm^2 (size of CP: 1.5 × 1.5 cm^2) and dried under ambient conditions.

Synthesis of Ni-HHTP-NF. The self-supported Ni-HHTP-NF was fabricated by a facile bath oiling approach. Typically, NF with the size of 1.5 × 1.5 cm^2 was firstly treated with 2.0 M HCl solution for 1 h and then washed with deionized water for 3 times and ethanol for 3 times. After drying at room temperature, the pre-treated NF was immersed into an autoclave containing HHTP (7 mmol), $Ni(CH_3COO)_2 \cdot 4H_2O$ (10 mg) in deionized water (4 mL). Then the autoclave was sealed and heated at 85°C for 12 h. The resulting Ni foam was washed with the mixture of water and ethanol (v/v= 1:1) for three times and finally dried in an ambient environment. Ni-HHTP was synthesized by the same procedure without NF.

Synthesis of Ni-BDC-NF. The self-supported Ni-BDC-NF was fabricated by the previous work, where NF was used as both the metal source and substrate.[1] Typically,

NF was firstly treated with 2.0 M HCl solution, and then washed with deionized water for 3 times and ethanol for 3 times. Then, the pre-treated NF was immersed into an autoclave containing 1.20 mmol H_2BDC, 25 mL DMA and diluted HCl solution (5.0 mL, 0.05 M). Then the autoclave was sealed and heated at 120°C for 20 h. After cooling, the resulting Ni foam was taken out, and then ultrasonically treated in deionized water for 1 min, washed with the mixture of water and ethanol solution (v/v= 1:1) for three times, and finally dried in the ambient environment.

Electrical Conductivity Measurement. As-synthesized Bi-HHTP crystalline powder was pelletized in a pelletizer of 3 mm diameter by a hydraulic press with a force of 8 MPa. The thicknesses of pellets were measured with an electronic digital caliper. I-V curves measurement was performed with a Keithley 4200 system in the air at room temperature. The voltage (V vs. counter) was scanned from -5.0 to 5.0 V at room temperature in the air. σ was calculated from the current (I) and voltage (V) data according to the equation $\sigma = (I/V) \times (d/S)$, where d is the sample thickness, and S is the cross-sectional area of the conductor.

Thermogravimetric analysis (TGA). Thermogravimetric analysis data were gathered on a sample of Bi-HHTP using a TA Instruments Discovery TGA. The sample was put into a platinum crucible and heated in the air from 28°C to 800°C with a heating rate of 10°C min^{-1}.

Electrochemical CO_2 Reduction Measurements. Electrochemical measurements were performed with a CHI 760E electrochemical workstation (Shanghai, Chenhua, China) using a three-channel flow cell comprising Bi-HHTP/CP as the gas diffusion layer (GDL), NF as a counter electrode, and Ag/AgCl (saturated KCl electrolyte) as a reference electrode. For electrochemical CO_2 reduction experiments, the anion exchange membrane separated the cathode and anode chamber (Fumasep, FAB-PK-130). And the electrolyte was 1 M KOH without special treatment. The electrolyte in the cathode and anode was circulated by a peristaltic pump (Kamoer, F01A-STP), with a flow rate of 10 mL min^{-1}. The high-purity CO_2 (99.9995%) gas was continuously passing through the flow chamber with the flow rate of 20 mL min^{-1} via a mass flow controller (Sevenstar, D07-7B). All potentials were measured versus an Ag/AgCl

reference electrode without iR compensation. The potentials were converted to the RHE scale with the following equation: E (versus RHE) = E (versus Ag/AgCl) + 0.197 + 0.059 × pH, and the presented current density was normalized to the geometric surface area ($1 \times 1\ cm^2$). All experiments were carried out at room temperature.

Product Quantification. The liquid products were quantified by ^{1}H NMR spectroscopy (Bruker-DRX 400 MHz) using dimethyl sulfoxide (DMSO) as an internal standard. The pre-saturation method was used to suppress the water peak. The FE was calculated using the following equation:

$$FE(\%) = \frac{96485\ (C\ mol^{-1}) \times n\ (mol\ mL^{-1}) \times N \times 20\ (mL\ min^{-1}) \times 60\ (min)}{Q} \times 100\%,$$

Where 96485 is the Faraday constant ($C\ mol^{-1}$), n is the number of products per milliliter ($mol\ mL^{-1}$), N is the electron transfer number (formate, CO, and H_2), 20 is the flow rate of CO_2 ($mL\ min^{-1}$), 60 is reaction time (min) and Q is the total charge obtained from chronoamperometry.

Calculation of cathodic energy efficiency. The cathodic energy efficiency (CEE) was calculated based on the cathodic CO_2RR coupled with an anodic oxygen evolution reaction (OER).

$$CEE_{\text{formate}} = (E^0_{\text{ox}} - E^0_{\text{CO2RR}}) / (E_{\text{ox}} - E_{\text{CO2RR}}) \times FE_{\text{formate}},$$

Where, E^0_{ox} and E^0_{CO2RR} are the thermodynamic potentials for OER (1.23 V vs. RHE) and CO_2RR to formate (-0.21 V vs. RHE), respectively. E_{ox} and E_{CO2RR} correspond to the applied potentials at the anode and cathode, respectively. For the calculation of the half-cell CEE, the overpotential of the anodic reaction was set as 0 V.

The formate generation was calculated according to the following formula:

$$\text{Formate generation} = C_{\text{formate}} \times V / (t \times M_{\text{formate}}),$$

Where C_{formate} was the measured concentration of formate in the solution. V represented the volume of electrolyte, M_{formate} was the molecular weight of formate

(45.02 g mol^{-1}), and t was on behalf of the electrolysis time (1 h).

Calculation of TOF values for catalyst. The calculation of turnover frequency (TOF) value was calculated using the following formula:[2]

$TOF = jA/2nN_Ae = j \times A / (m_{cat} \times 2 \times n \times F / M_{cat})$,

The current density j (mA cm^{-2}) for formate at specific potential (vs. RHE) was chosen to calculate the amount of charge each metal site can provide, and the electrode surface area A was about 1 cm^2. The current required to convert one formate molecule is $2e^-$. F is Faraday's constant (96485 C mol^{-1}), and n is metal loading (%) in the catalyst. M_{cat} is the atomic mass of metal, which is 114.82 g mol^{-1} for In and 208.98 g mol^{-1} for Bi. On the working electrode, the mass (m) of the metal is 1 mg. The actual proportion of elements in the material was determined by ICP-OES (Bi: 59.93%; In: 21.06%). The result of In content was close to the data of EDS of In-HHTP.

Double-layer capacitance (C_{dl}) measurement. Cyclic voltammogram measurements of the catalysts were conducted from -0.6 to -0.8 V vs. RHE with various scan rates to obtain the double-layer capacitance (C_{dl}) of different catalysts. The C_{dl} was estimated by plotting the Δj (j_a - j_c) at -0.7 V vs. RHE against the scan rates, in which j_a and j_c are the anodic and cathodic current densities, respectively. The linear slope was equivalent to the C_{dl}.

Table S1. Crystal data and structure refinement of Bi-HHTP.

Identification code	Bi-HHTP
Empirical formula	$C_{36}H_{20}Bi_4O_{16.3}$
Formula weight	1549.16
Temperature/K	159.99(10)
Crystal system	monoclinic
Space group	$P2_1/n$
a/Å	16.9776(17)
b/Å	14.3793(4)
c/Å	26.623(3)
α/°	90
β/°	143.57(2)
γ/°	90
Volume/Å^3	3859.8(13)
Z	4
ρ_{calc}g/cm^3	2.666
μ/mm^{-1}	35.795
F(000)	2793.0
Crystal size/mm^3	0.076 × 0.032 × 0.027
Radiation	Cu Kα (λ = 1.54184)
2Θ range for data collection/°	5.406 to 151.242
Index ranges	$-21 \le h \le 20$, $-17 \le k \le 17$, $-32 \le l \le 33$
Reflections collected	50191
Independent reflections	7747 [R_{int} = 0.0441, R_{sigma} = 0.0291]
Data/restraints/parameters	7747/25/522
Goodness-of-fit on F^2	1.025
Final R indexes [I>=2σ (I)]	R_1 = 0.0612, wR_2 = 0.1756
Final R indexes [all data]	R_1 = 0.0664, wR_2 = 0.1804
Largest diff. peak/hole / e Å^{-3}	6.95/-1.85

Table S2. The conductivity of different MOFs.

Materials	Electrical conductivity (S m^{-1})	Ref.
Bi-HHTP	1.65	This work
Cu-HHTP	5.1×10^{-5}	3
Cu-DBC	1.2×10^{-2}	4
MIP-202(Zr)	1.1	5
$Fe_2(BDP)_3$	0.96	6
Cd_2(TTFTB)	2.86×10^{-2}	7
$[Zn(H_2PO_4)_2(C_2N_3H_3)_2]n$	2×10^{-3}	8
Zn-HAB	8.6×10^{-2}	9
Cu-THQ	5.4×10^{-6}	10
NiCB@NU-1000	2.7×10^{-5}	11
TCNQ@Cu_3BTC_2	7	12
Co_3HITP_2	0.08	13
Ni_3HITP_2	189.6	13
$Cu_3(BHT)_2$	158000	14

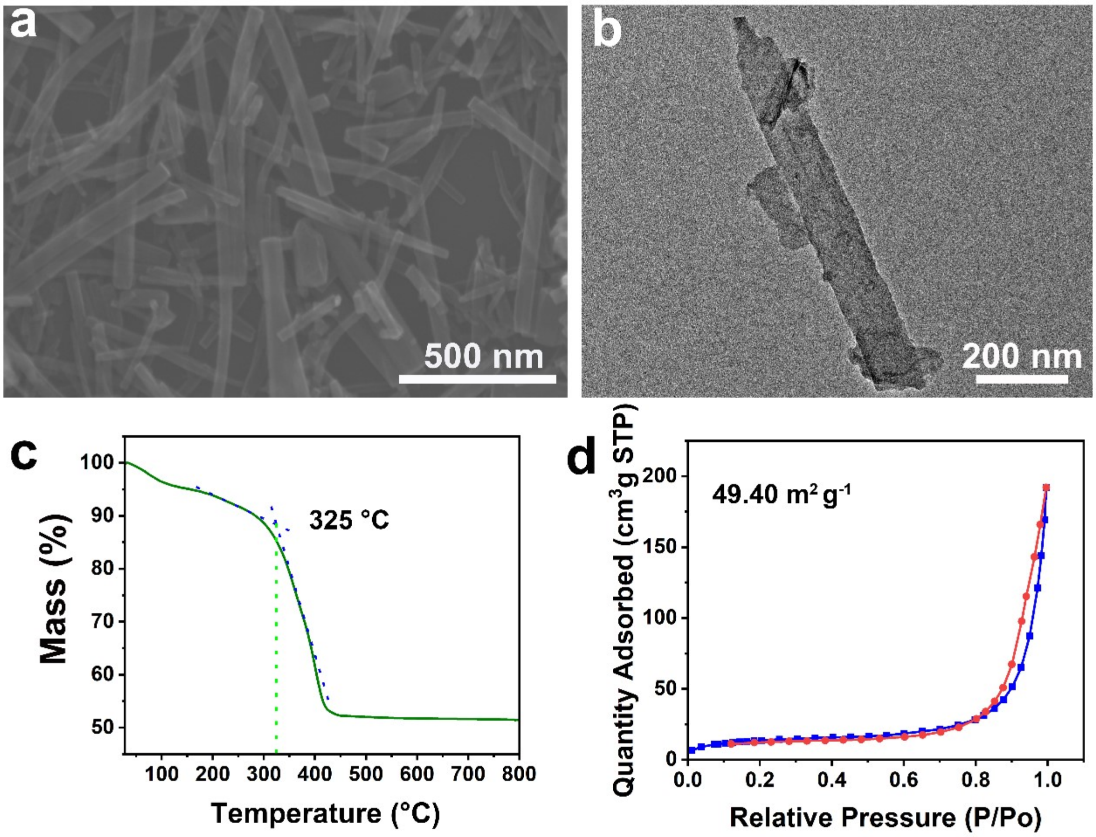

Fig. S1. (a) SEM and (b) TEM images of Bi-HHTP. (c) TGA and (d) BET analysis of Bi-HHTP.

The thermal stability of Bi-HHTP was measured by TGA in Fig. S1c, and Bi-HHTP exhibited a high temperature (325°C). Subsequently, the specific surface area of Bi-HHTP was then structurally characterized using Brauner-Emmet-Teller (BET) analysis utilizing N_2 adsorption and desorption isotherms obtained on a Micromeritics 3FLEX instrument, and the BET of Bi-HHTP is 49.40 $m^2 g^{-1}$ (Fig. S1d).

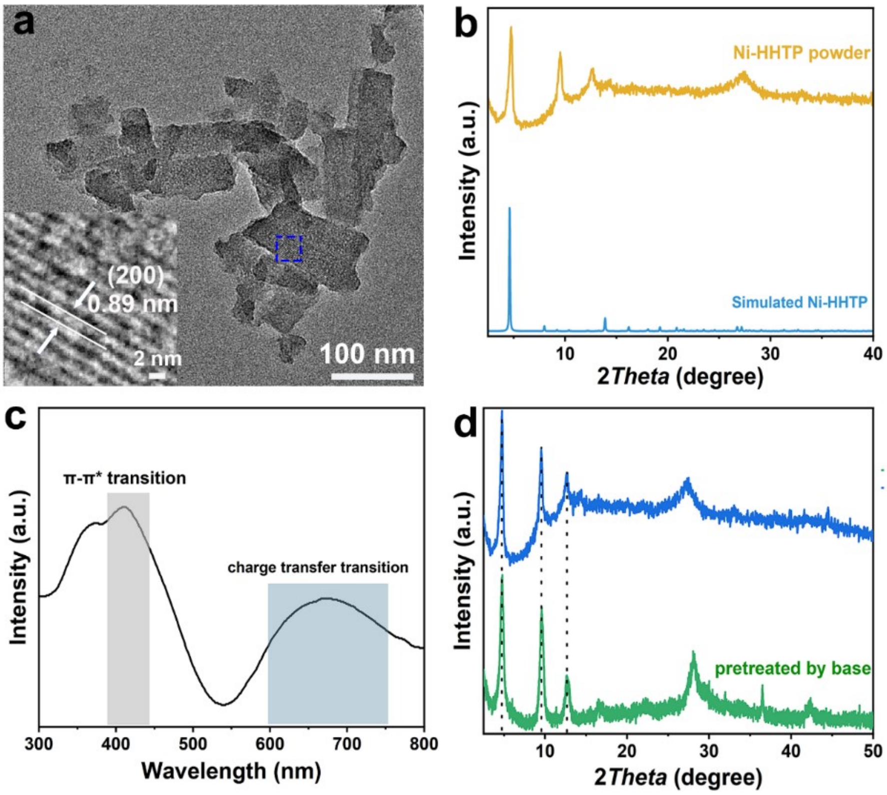

Fig. S2. (a) TEM image, (b) XRD patterns, and (c) UV-vis spectrum of as-synthesized Ni-HHTP. (d) XRD patterns of Ni-HHTP before and after immersing in 1 M KOH aqueous solution.

In our work, besides Bi-HHTP, Ni-HHTP, and In-HHTP were also synthesized to investigate the relationship between metal elements and performance. TEM image indicates that Ni-HHTP shows a nanorod-like morphology (Fig. S2a), and the uniform pillar structure and lattice spacing of 0.89 nm shown in the HRTEM reveal the prominent crystal characteristics of Ni-HHTP (inset of Fig. S2a). The XRD result further certifies the crystal characteristics of Ni-HHTP (Fig. S2b). The peaks at ~380

nm and ~700 nm in UV-vis spectrum of Ni-HHTP respond to π-π* and LMCT, respectively (Fig. S2c). The stability of Ni-HHTP was also evaluated by subjecting the material to the KOH aqueous solution (Fig. S2d), and the result demonstrated that it remained intact even in the strong base. Similarly, TEM image shows that In-HHTP displays a nanorod-like morphology (Fig. S3a). Moreover, the structure of In-HHTP was similar to that of Ni-HHTP (Fig. S3b). The EDS result in Fig. S3c shows that In ions account for 25.56% of the total structure. Meanwhile, the uniform distribution of the In, C, and O elements throughout the whole structure was shown in the corresponding element mapping (Fig. S3d).

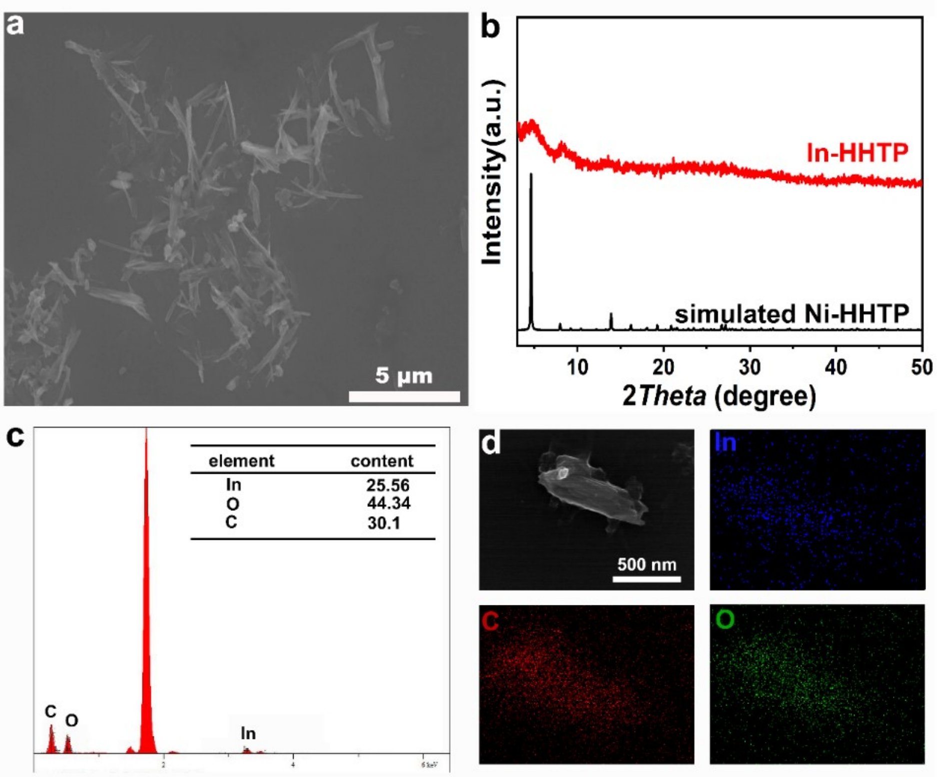

Fig. S3. (a) SEM image of In-HHTP. (b) XRD pattern of In-HHTP. (c) EDS pattern of In-HHTP. (d) HAADF-STEM and the corresponding elemental mappings of In-HHTP.

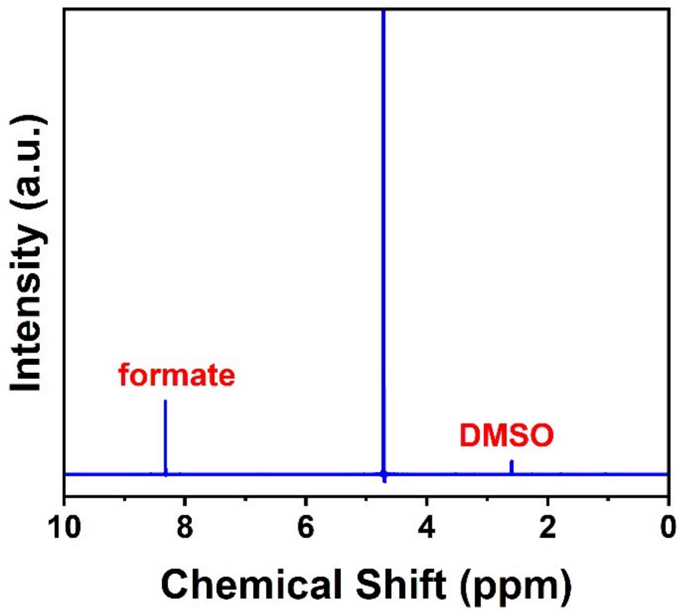

Fig. S4. The ^{1}H-NMR spectrum of liquid products.

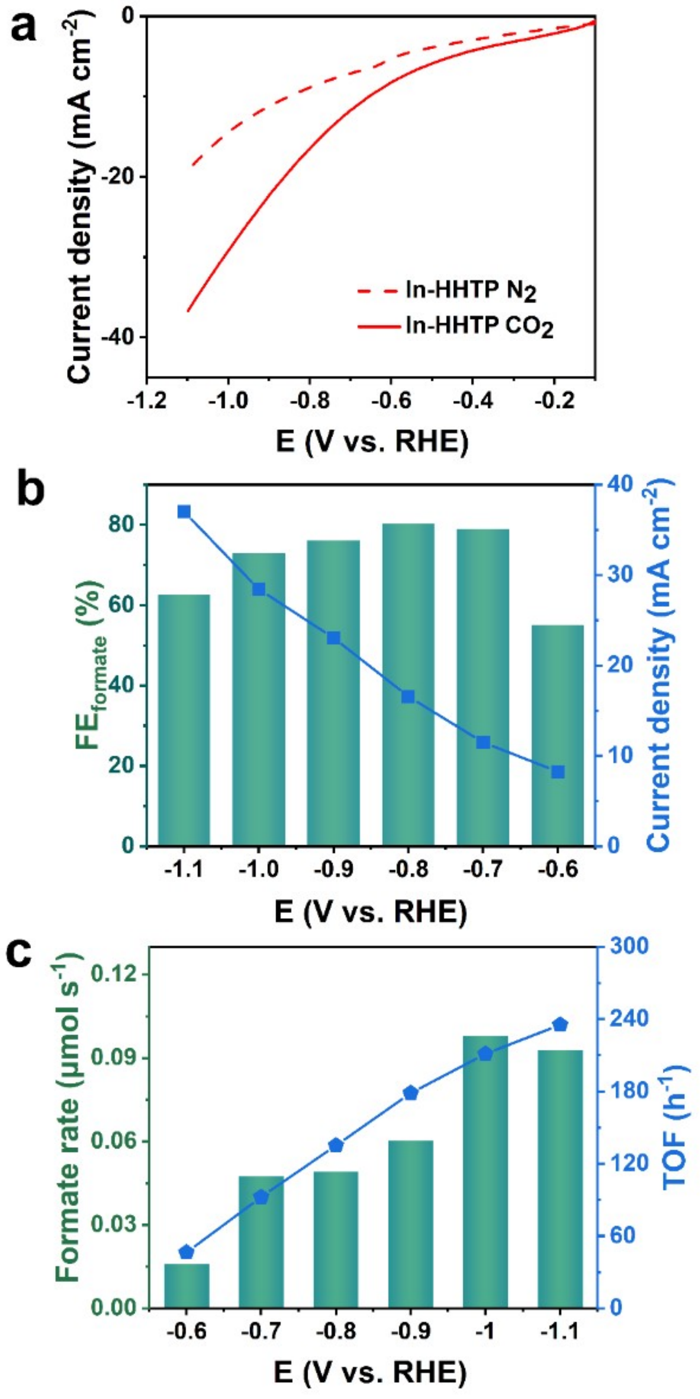

Fig. S5. (a) LSV curves of In-HHTP in CO_2 or N_2 atmosphere without correction. (b) Potential-dependent FEs for formate and current densities of In-HHTP. (c) Generation rates of formate and TOF of In-HHTP at different potentials.

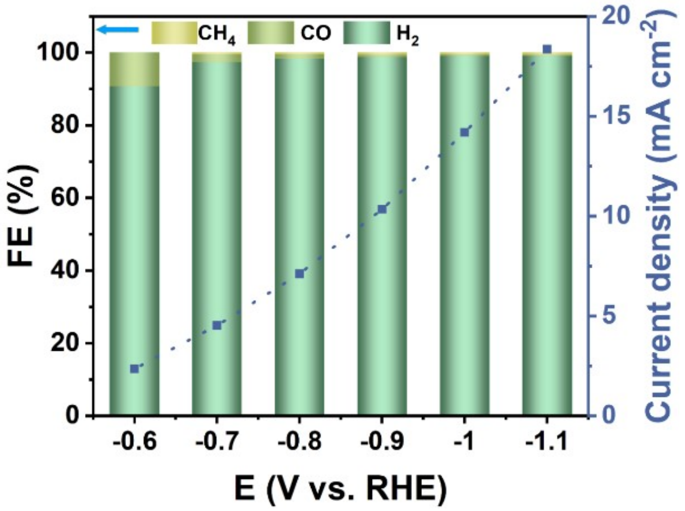

Fig. S6. Potential-dependent FEs and current densities of Ni-HHTP.

As illustrated in Fig. S5 and S6, the electrocatalytic performances of In-HHTP and Ni-HHTP towards CO_2RR were also tested. Firstly, In-HHTP exhibited a more positive onset potential and dramatically larger current densities than those in the N_2-purged electrolytes, signifying that the In-HHTP could also lower the reaction barriers for electrocatalytic CO_2RR (Fig. S5a). The current density of In-HHTP was around 40 mA cm^{-2} at -1.1 V vs. RHE (Fig. S5b) and the best $FE_{formate}$ was 80%, indicating its worse performance compared to Bi-HHTP. The TOF of Bi-HHTP was approximately three times that of In-HHTP (Fig. S5c). The production rate of formate for Bi-HHTP was five times greater than that for In-HHTP at -1.1 V vs. RHE. However, Ni-HHTP showed poor performance towards electrocatalytic CO_2RR (Fig. S6), in terms of current density and selectivity (the main product was H_2).

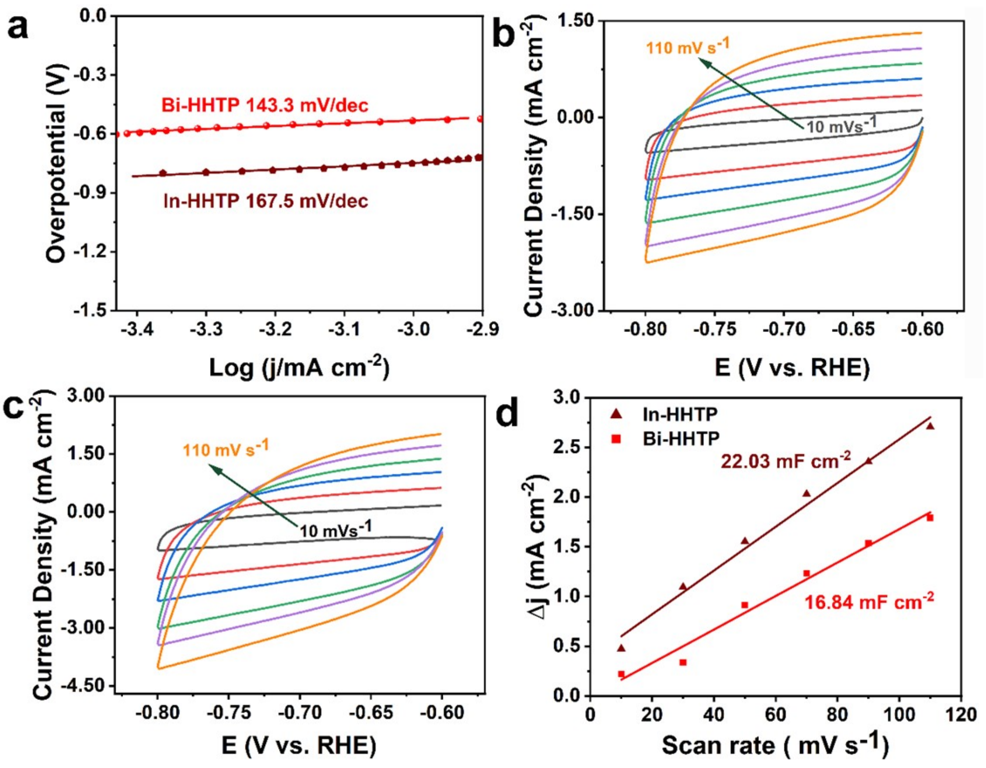

Fig. S7. (a) Tafel analysis of Bi-HHTP and In-HHTP. Cyclic voltammetry (CV) data of (b) Bi-HHTP and (c) In-HHTP at the non-Faradaic region with various scan rates from 10 mV s^{-1} to 110 mV s^{-1}. (d) The data of double-layer capacitances (C_{dl}) for Bi-HHTP and In-HHTP.

To initially explore the mechanism in various electrodes on the chemical rate-determining step (RDS), Tafel analysis was first tested. The Tafel slope of 143.3 mV/decade for Bi-HHTP (Fig. S7a) was comparable to the 118 mV/decade, which accounted for rate-limiting single-electron transfer from the adsorbed CO_2 to generate the surface adsorbed $CO_2{}^{\cdot -}$ and smaller Tafel slope indicates the better catalytic performance.[15] The electrical double-layer capacitance (C_{dl}) acquired by the non-Faradaic region with various scan rates (Fig. S7b and S7c) was further applied to calculate the electrochemically active surface area (ECSA). The smaller C_{dl} of the Bi-HHTP electrode (16.84 mF cm^{-2}) in Fig. S7d exhibited a more positive potential, indicating the higher intrinsic catalytic activity towards CO_2RR.

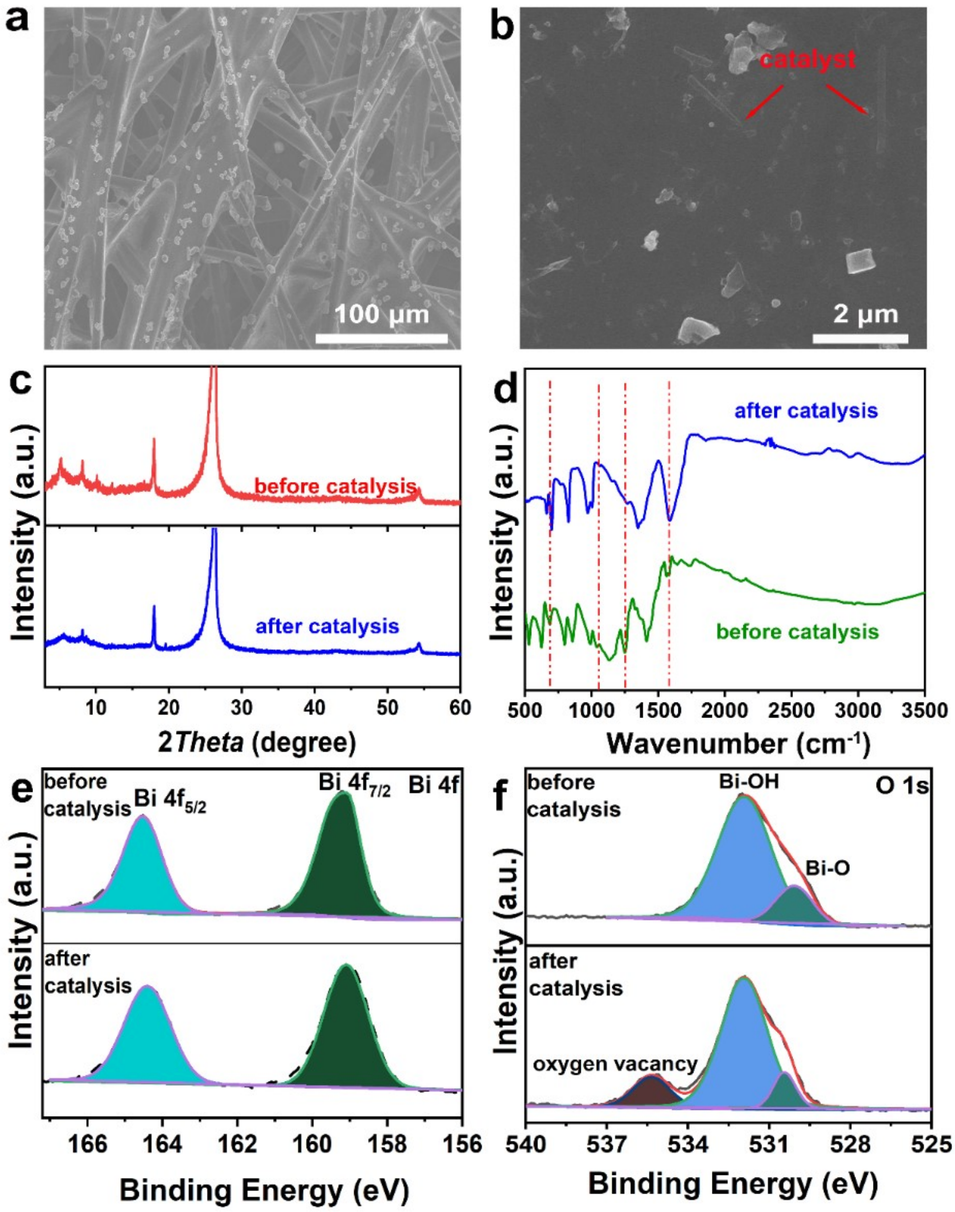

Fig. S8. (a) SEM image of Bi-HHTP/CP before catalysis. (b) SEM image of Bi-HHTP after catalysis. (c) XRD patterns and (d) FT-IR spectra of Bi-HHTP before and after catalysis. High-resolution (e) Bi 4f and (f) O 1s XPS spectra of Bi-HHTP before and after electrolysis.

Given the unique nanostructure, Bi-based MOFs were usually employed as excellent pro-electrocatalysts, generating the metallic Bi for CO_2RR via the cathodic *in situ* reductions.[16] In response to the question of whether reduction and reconstruction occurred in Bi-HHTP during CO_2RR, a series of structural characterizations on catalysts were conducted. Firstly, as shown in Fig. S8a and S8b, the morphology of Bi-

HHTP did not undergo significant change, compared with that before the catalysis. XRD results showed that no diffraction character of metal Bi substance (No. 44-1246) existed after excluding the signals of CP (Fig. S8c), indicating that Bi-HHTP was indeed stable in the process of CO_2RR. The FTIR spectra of Bi-HHTP further demonstrated that the characteristic peak of Bi-HHTP did not disappear and that the Bi-O vibration existed after electrolysis, which also certified the maintaining topology of Bi-HHTP (Fig. S8d). Furthermore, XPS was further performed to shed light on the surface electronic states of the elements before and after electrolysis, where the binding energies at 159.2 and 164.5 eV could be assigned to Bi $4f_{7/2}$ and Bi $4f_{5/2}$ of Bi^{3+}, respectively.[17] The Bi peak of reacted Bi-HHTP did not shift, compared with the pristine counterpart (Fig. S8e). Moreover, the binding energies of O 1s at 530.1 and 531.8 eV in Fig. S8f were respectively associated with the lattice oxygen (Bi-O) and chemically adsorbed oxygen (Bi-OH), where the chemically adsorbed oxygen dominated in Bi-HHTP.[18] The obvious peak that occurred at 535.4 eV after the reaction may be attributed to oxygen vacancy, implying that a large number of defects were further produced during the reconstruction of catalysts. Note that the coordinatively unsaturated metal sites could effectively promote ion transport,[19] which is beneficial to CO_2RR.

Table S3. The performance of different catalysts for the conversion of CO_2 to formate.

Catalyst	Cell Type	Electrolyte	Current density (mA cm^{-2})	Faradaic efficiency (%)	Potential (V *vs.* RHE)	Ref.
Bi-HHTP	flow cell	0.1 M $KHCO_3$	93 at -1.1 V vs. RHE	95	-0.8	This work
Bi-NHS	H cell	0.5 M $KHCO_3$	~21	96	-0.96	[20]
Bi-enes	H cell	0.5 M $KHCO_3$	70 at -1.178 V vs. RHE	Nearly 100	-0.978	[21]
Bi-D	flow cell	1 M KOH	136 at -1 V vs. RHE	94.6	-0.8	[22]
BiNSs	H cell	0.5 M $KHCO_3$	62	93	-0.95	[23]
Bi-ene	flow cell	1 M KOH	200	close to 100	-0.75	[24]
SU-101 NRs	flow cell	0.5 M $KHCO_3$	45.85	88.85	-1.06	[25]
Bi-MOF	H cell	0.1 M $KHCO_3$	15 at -1.1 V vs. RHE	92.2	-0.9	[26]
Bi-OAm-300	H cell	0.5 M $KHCO_3$	32.1	97.1	-0.9	[27]
Bi-Sn aerogel	H cell	0.1 M $KHCO_3$	18.4	93.9	-1	[28]
Bi NS	H cell	0.1 M $KHCO_3$	17 at -1.3 V vs. RHE	92	-1.1	[29]
Bi-ene-NW	flow cell	0.5 M $KHCO_3$	85 at -1.17 V vs. RHE	95	-0.9	[30]
Bi(btb)	flow cell	0.5 M $KHCO_3$	About 11	95	-0.97	[31]
Bi-NRs@NCNTs	H cell	0.1 M $KHCO_3$	6	90.9	-0.9	[32]
Cufoam@BiNW	H cell	0.5 M $NaHCO_3$	46	88	-1.09	[33]
NTD-Bi	flow cell	1.0 M KOH	288	98	-0.6	[34]
BBNs	H cell	0.5 M $KHCO_3$	13.5	91	-0.85	[35]
Bi-Sn/CF	H cell	0.5 M $KHCO_3$	53	96	-1.14	[35]
MIL-68(In)-NH_2	flow cell	1.0 M KOH	108	94.4	-1.1	[36]
Sulfur-doped indium	H cell	0.5 M $KHCO_3$	86 at -1.23 V vs. RHE	93	-0.98	[37]

The conventional electrochemical CO_2 conversion system includes the oxygen evolution reaction (OER) and CO_2RR. However, the OER with high thermodynamic potentials and slow kinetics could consume about 90% of the power input, which reduced the economic efficiency of this system. Furthermore, compared to OER, the thermodynamically favorable methanol oxidation reaction (MOR) can significantly diminish the power input with fast kinetics and positive potentials to reduce the energy consumption in the overall system. The Ni-based metal-organic frameworks (MOFs) with permanent porosity and the exposed metal sites showed a prominent performance for MOR.[1] However, there is still little research on conductive MOF electrocatalysts for efficient MOR. Thus, the conductive MOF (Ni-HHTP) grown on Ni foam via the traditional solvothermal process was investigated in a flow-type cell.

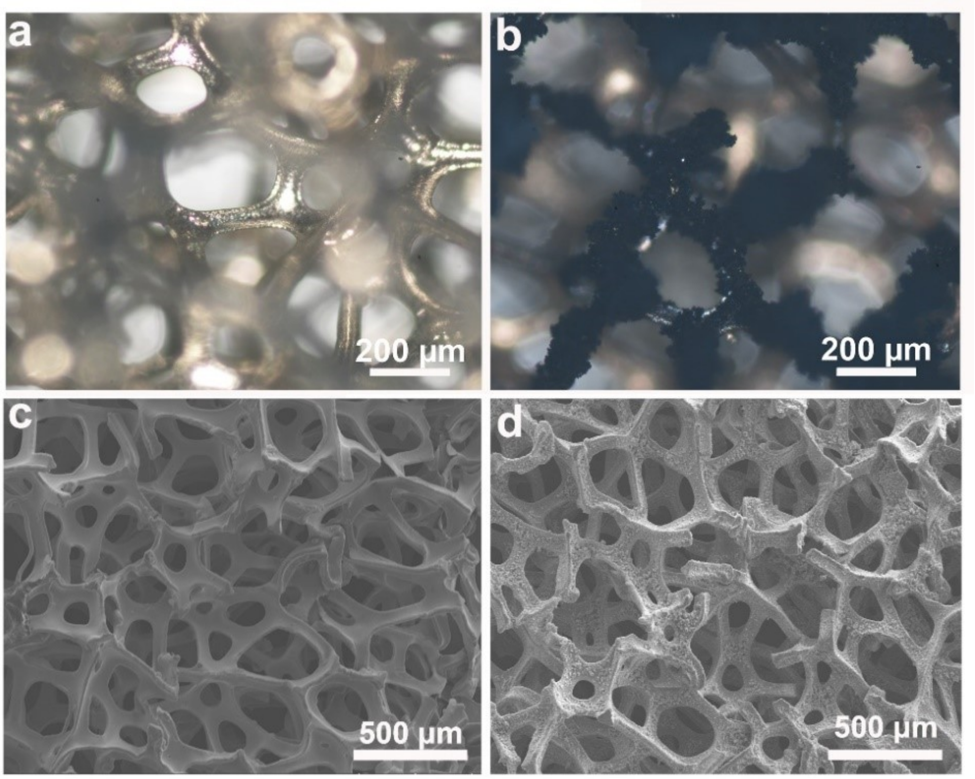

Fig. S9. The optical microscope (OM) and SEM images. (a) and (c): Ni foam; (b) and (d): Ni-HHTP-NF.

As shown in Fig. S9, the OM and SEM images show that the surface of the pre-treated NF was smooth (Fig. S9a and S9c). Subsequently, an obvious dendrite on the NF was observed after the growth of Ni-HHTP (Fig. S9b and S9d). The morphology of Ni-HHTP on NF is nanorod-like with a size of ~200 nm (Fig. S10a and S10b). The EDS data in Fig. S10c displayed that the Ni element only accounted for 6.96 wt% on the surface of materials, and all elements (C, O, Ni) are evenly distributed on the surface of Ni-HHTP-NF (Fig. S10d).

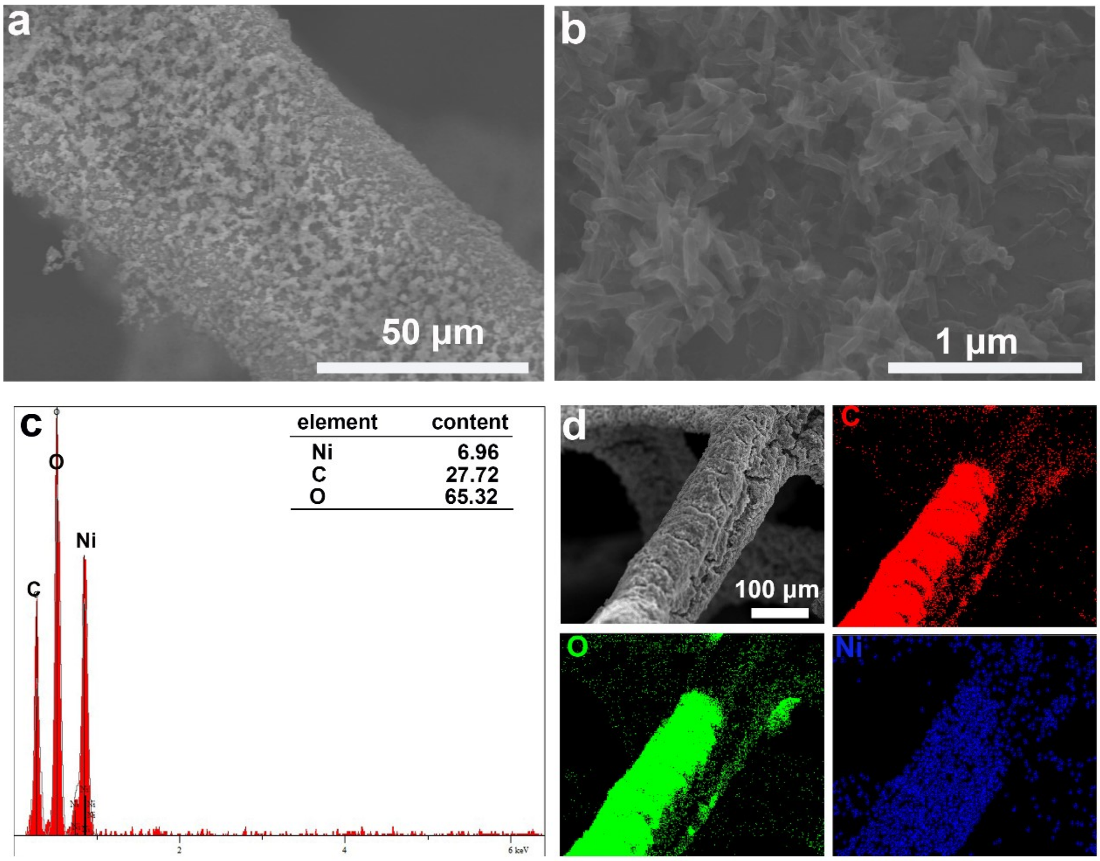

Fig. S10. (a) Low-magnification and (b) high-magnification SEM images of Ni-HHTP-NF. (c) EDS pattern. (d) SEM and EDS elemental mapping of Ni-HHTP-NF.

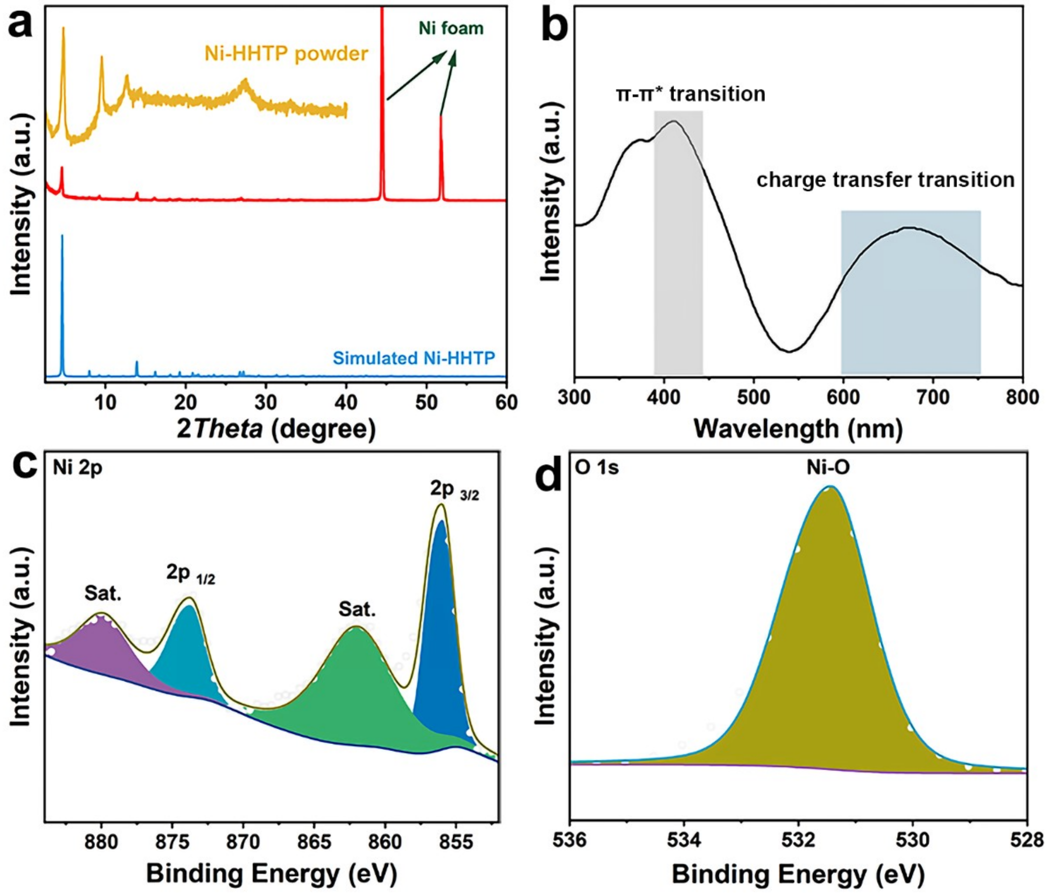

Fig. S11. (a) XRD pattern, (b) UV-vis spectrum, high-resolution (c) Ni 2p and (d) O 1s XPS spectra of Ni-HHTP-NF.

The XRD pattern of Ni-HHTP-NF is consistent with the simulated one and the as-prepared MOF powder, confirming its high purity (Fig. S11a).[38] Meanwhile, the UV-vis spectrum in Fig. S11b further showed the characteristic electronic absorption bands of Ni-HHTP at ~380 and ~700 nm corresponding to the distinct peaks of π-π* and transition LMCT, respectively, indicating the formation of coordination bonds between Ni^{3+} ions and HHTP. The XPS of Ni 2p in Fig. S11c was subsequently examined to achieve a better understanding of the surface features. The Ni 2p spectrum may be randomly divided into two distinct peaks at 856.2 eV and 873.7 eV attributed to Ni $2p_{3/2}$ and Ni $2p_{1/2}$ spin-orbits, respectively. Nevertheless, the peaks at 861.0 eV and 880.8 eV corresponding to the satellite peaks of Ni $2p_{3/2}$ and Ni $2p_{1/2}$ respectively indicated the existence of Ni^{2+}.[39, 40] The O 1s spectrum in Fig. S11d showed the peak locked at 531.4

eV, related to the Ni-O bond between hydroxide groups and Ni ions.[41]

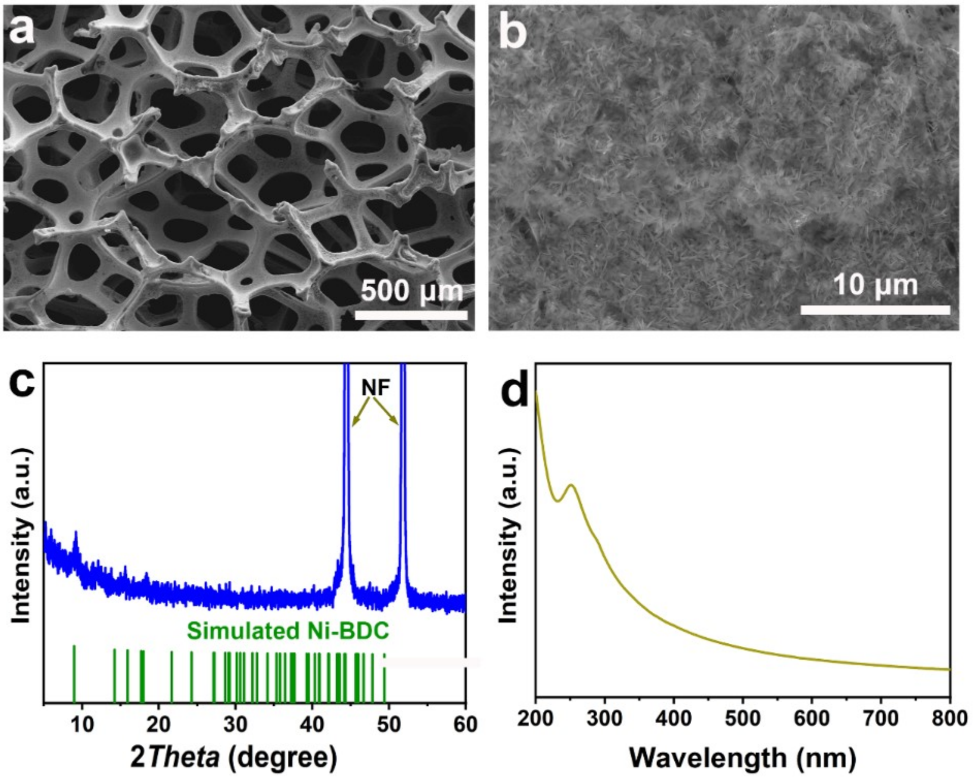

Fig. S12. (a) SEM image of Ni-BDC-NF at low magnification. (b) SEM image of Ni-BDC-NF at high magnification. (c) XRD pattern and (d) UV-vis spectrum of Ni-BDC-NF.

As a comparison, we also synthesized Ni-BDC-NF via the reported method,[44] and its characterization was displayed in Fig. S15. SEM images in Fig. S12a and S12b show that the Ni-BDC displays a nanosheet-like morphology, where Ni-BDC is uniformly distributed on the surface of NF by layer-by-layer stacked arrays. XRD pattern of Ni-BDC-NF in Fig. S12c is well consistent with that of the reported, further indicating that Ni-BDC is successfully grown on NF.[42, 43] A strong absorption peak occurred at around 200 to 400 nm in the UV-vis spectrum (Fig. S12d), attributed to the transfer of the charge from the oxygen center of the organic bridge to the metal center.[44]

It was reported that Ni^{2+} species can be transformed into Ni^{3+} species (NiOOH) during electrooxidation reactions, which were considered as the real active species.[45, 46] But recent literature reports that electrophilic oxygen species (O* or OH*) formed

on the electrocatalyst are considered to be the catalytically active species.[47] That means more stable electrocatalysts can be utilized in this field and nickel non-hydroxides could exhibit better performance. *In situ* Raman spectroscopy was used to investigate the changes in the catalyst during the OER process. The *in situ* Raman spectrum of Ni-HHTP-ET for OER under different potentials (1 M KOH electrolyte) was shown in Fig. S13. In the OER process, the broad peak observed in the 400 - 600 cm^{-1} wavenumber region was attributed to the formation of $NiOO^-$ at high potentials (~1.97 V).[48] No signal peaks corresponding to the bending vibration and stretching vibration of Ni^{3+}-O of γ-NiOOH appeared at ~1.37 V, and Ni species were usually oxidized in this potential. *In situ* Raman spectroscopies illustrated that the Ni-HHTP possessed better chemical stability towards potentials.

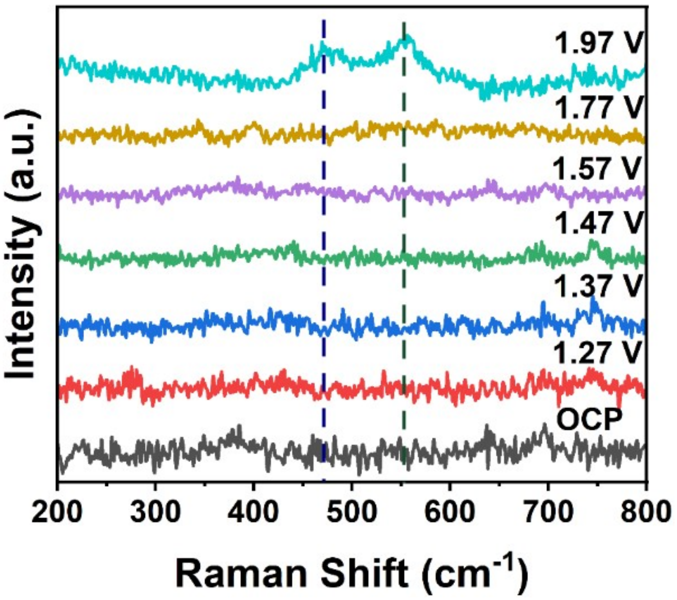

Fig. S13. *In situ* Raman spectroscopies of Ni-HHTP-ET for OER under different potentials (1 M KOH).

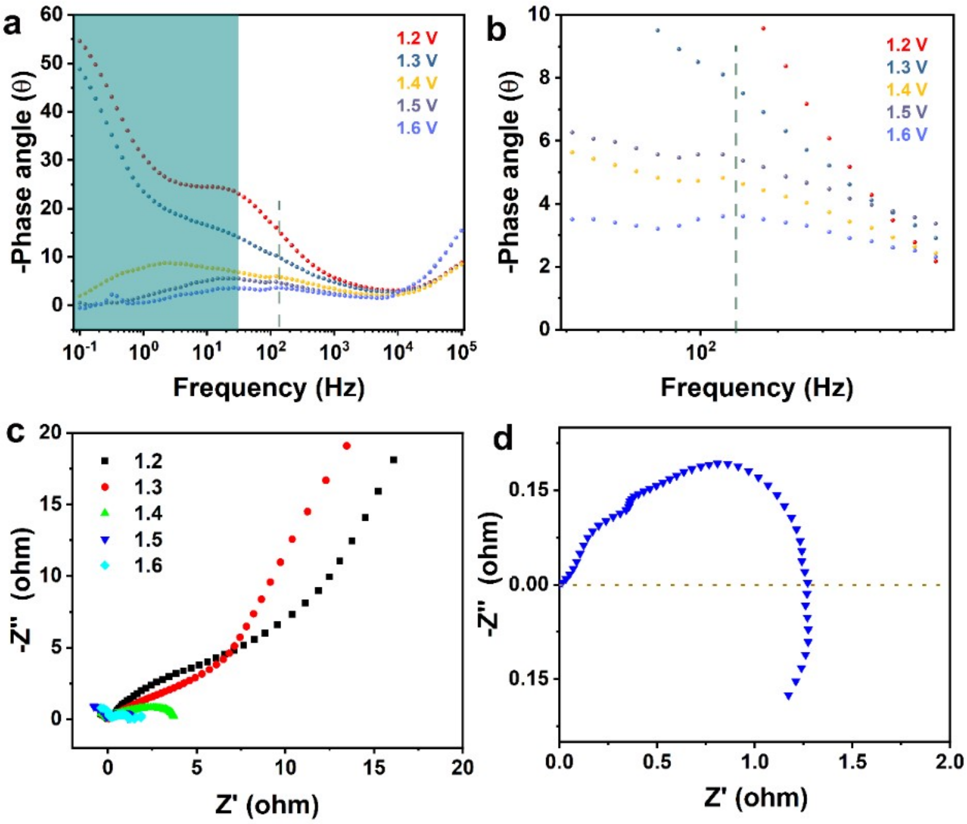

Fig. S14. (a, b) Bode plots. (c) Nyquist plots for MOR at different potentials. (d) Nyquist plots for MOR at 1.5 V vs. RHE.

The electrochemical processes of MOR were then tracked by operando electrochemical impedance spectroscopy (EIS) at different potentials. The response appearing in the low-frequency region may result from the nonhomogeneous charge distribution caused by surface oxidized species. Obviously, one response signal was displayed in the frequency region around 100 Hz in the Bode plot and no other phase angle peak occurred in low frequency and higher frequency (Fig. S14a and S14b), indicating that the catalyst was not electro-oxidized and MOR only occurred between the diffuse double layer and the surface of the catalyst.[47] Only a semicircle corresponding to the MOR reaction was shown after 1.4 V in the Nyquist plot (Fig. S14c). Interestingly, the real part at low frequency was contracted at ~1.5 V (Fig. S14d), attributed to the adsorption of electroactive species.

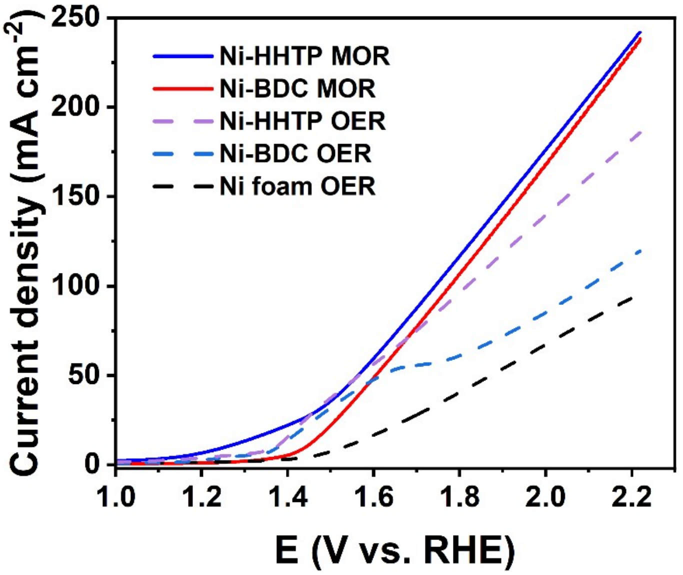

Fig. S15. MOR and OER polarization curves of Ni-HHTP-ET, and Ni-BDC-ET as the control sample.

The performances of Ni-BDC-ET and Ni-HHTP-ET towards OER and MOR were estimated in this work (Fig. S15). However, both the Ni-HHTP-ET and Ni-BDC-ET exhibited a better current density and a positive potential for MOR, compared to that for OER. The Ni-HHTP-ET exhibited a better performance towards MOR with positive potentials, compared to Ni-BDC-ET. The high surface area could provide a significant number of interfaces between GDL and electrolyte, providing better electrochemical activity and kinetics.[45] The ECSA was then estimated by the C_{dl} based on CVs recorded at different scan rates in the non-faradaic region from 0.5 - 1 V vs. RHE (Fig. S16). As shown in Fig. S16a and S16b, the current densities increased with increasing scan rates over Ni-HHTP-ET and Ni-BDC-ET. The higher C_{dl} of Ni-HHTP-ET (13.84 mF cm^{-2}) illustrated that the Ni-HHTP-ET could provide more effective active sites for MOR, compared to Ni-BDC-ET (5.94 mF cm^{-2}). The higher C_{dl} may also attribute to the characteristic organic ligand (HHTP) which could form a large π-π conjugated system and enhance ion transport or promote the penetration of electrolytes and strong adsorption of ions.

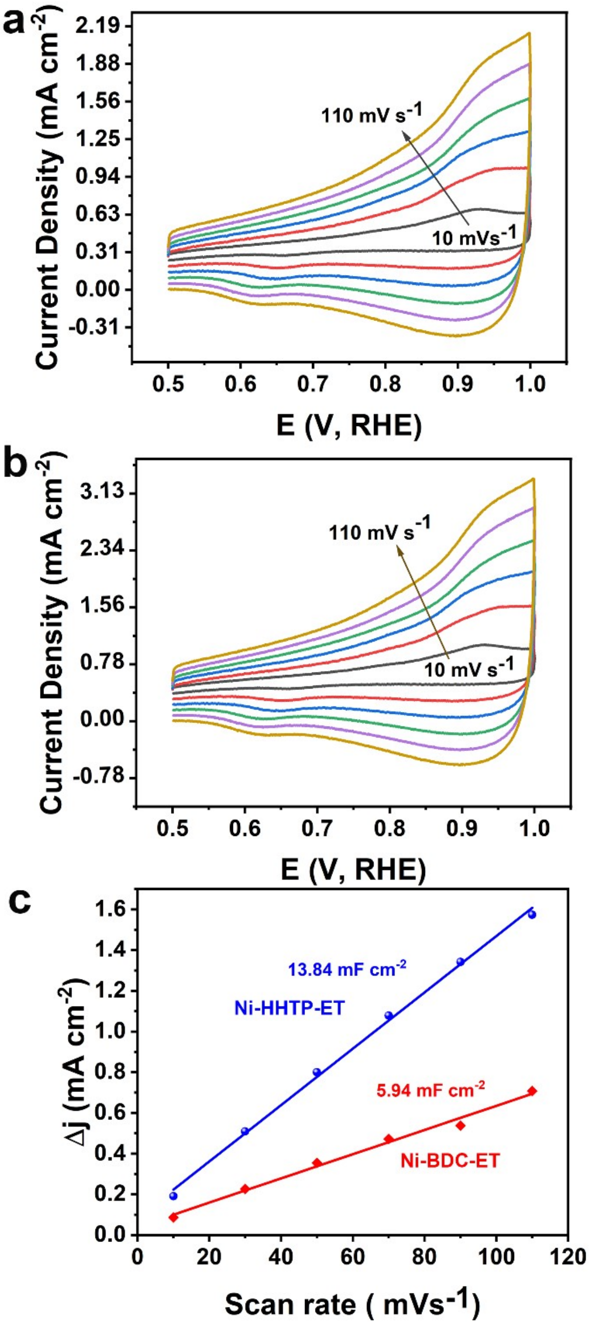

Fig. S16. Cyclic voltammetry (CV) data of (a) Ni-BDC-ET and (b) Ni-HHTP-ET at the non-Faradaic region with various scan rates from 10 mV s^{-1} to 110 mV s^{-1}. (c)The C_{dl} of different Ni-HHTP-ET and Ni-BDC-ET electrodes.

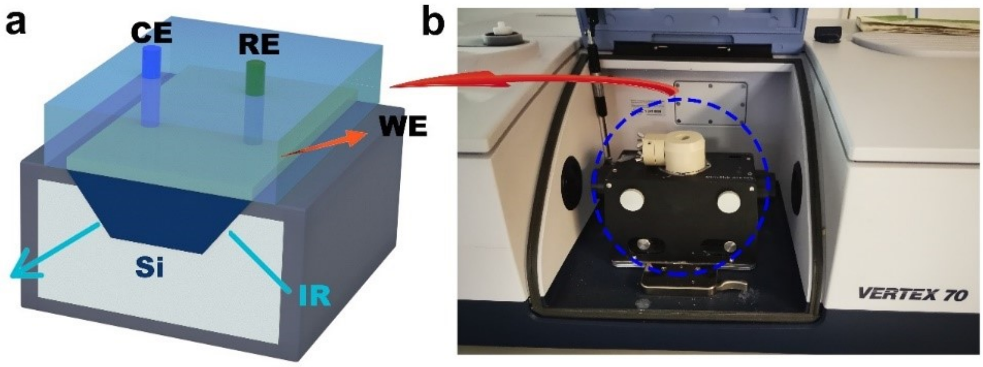

Fig. S17. (a) Illustration of the *in situ* ATR-FTIR system used for *in situ* characterization (WE, working electrode; CE, count electrode; RE, reference electrode. WE substrate is gold, and silicon prism (Si) is the reflection channel of IR). (b) The photo of the whole test system.

In situ FTIR spectroscopy as a powerful tool was also employed to explore the mechanistic studies of MOR in Ni-HHTP-ET electrodes by the detection of surface radicals and reaction intermediates using a commercial *in situ* attenuated total reflectance Fourier transform infrared spectroscopy (ATR-FTIR) device (Fig. S17) and providing information about characteristic vibrations of molecules on surfaces during the reaction. As shown in Fig. S18a, from the onset of the reaction at 1.35 V vs. RHE, some bands appeared in the spectra. The band at 1382 cm^{-1} was assigned to δ(CH) or ρr(COO) of $HCOO^-$/$HCOOCH_3^-$, and the band at 1254 cm^{-1} was assigned to ν(C-O) of formate.[49, 50] The band at 1640 cm^{-1} can be assigned to antisymmetric stretching vibration bands of OCO.[51] Furthermore, no evidence was found of the presence of either adsorbed CO species (bridge-bonded CO or linearly-bonded CO, 1700-2000 cm^{-1}) in the *in situ* ATR-FTIR (Fig. S18b).[50, 52] Besides, the band of CO_3^{2-} at around 1400 cm^{-1} can be found, indicating that further oxidation after the formation of formate may also occur in this reaction.[47]

To elucidate the underlying reason for the activity of Ni-HHTP-ET for MOR, density functional theory (DFT) calculations were performed. The Gibbs free energy profiles for the MOR process on Ni-HHTP are illustrated in Fig. S18c. The whole process follows (I-VII step): CH_3OH adsorption (II), CH_3O^*, CH_2O^*, CHO^*, $^*HCOOH$, $COOH^*$

adsorption and HCOOH generation. It can be seen that the potential-determining step for MOR is all contributed by the process of $^{*}CH_3OH \rightarrow {}^{*}CH_3O + H^+ + e^-$. VII is a competition reaction, with HCOOH working as the main production, which is consistent with the result of the band at 1382 cm^{-1} assigned to δ(CH) or ρr(COO) of $HCOO^-/HCOOCH_3^-$, the band at 1254 cm^{-1} assigned to v(C-O) of formate, and the band at 1640 cm^{-1} assigned to antisymmetric stretching vibration bands of OCO, as confirmed by *in situ* ATR-FTIR.

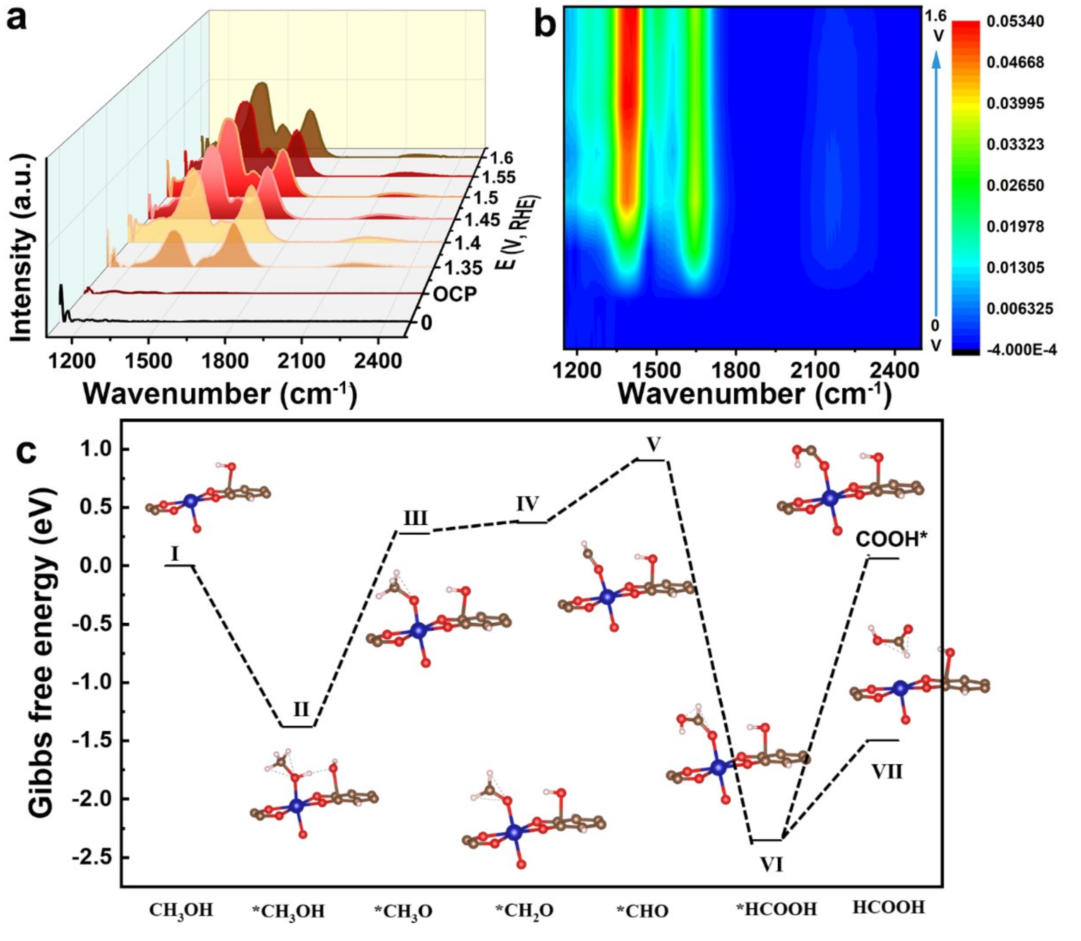

Fig. S18. (a) *In situ* ATR-FTIR spectra at different potentials of Ni-HHTP-ET during MOR. (b) Planar graph of *In situ* ATR-FTIR spectra. (c) Gibbs free energy diagram of MOR occurring on Ni-HHTP with blue, brown, red, and white balls representing Ni, C, O, and H.

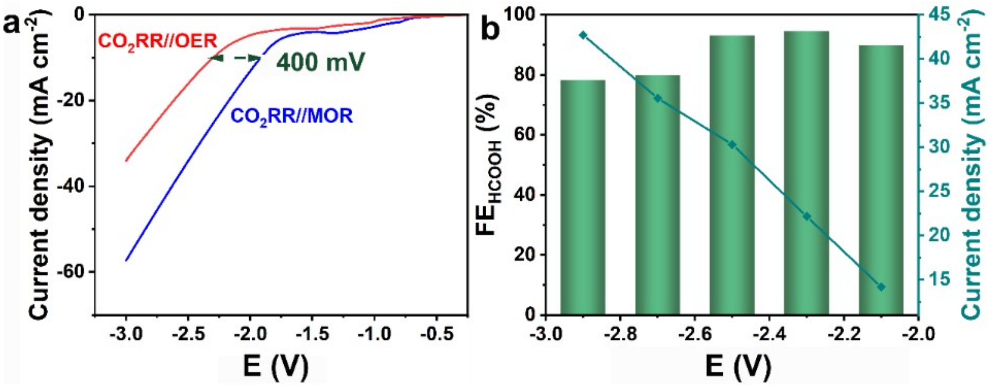

Fig. S19. (a) LSV curves of the constructed CO_2RR//MOR full cell based on the Bi-HHTP//Ni-HHTP-ET. (b) Voltage-dependent $FE_{formate}$ and current densities of Bi-HHTP.

Theoretical results:

Density functional theory (DFT), as implemented in the Vienna ab initio simulation program (VASP),[53, 54] served as the foundation for all theoretical computations. In the Generalized gradient approximations Perdew-Burke-Ernzerhof functional (GGA-PBE),[55, 56] the electron exchange and correlation energy was handled inside the generalized gradient approximation. The valence orbitals of Bi ($5d_{10}$, $6s_2$, $6p_3$), O (2s, 2p), and C (2s, 2p) were described by plane-wave basis sets with cutoff energies of 400 eV. The Monkhorst-Pack technique was used to produce the k-point sampling. The conjugate gradient (CG) approach completely relaxes the atomic coordinates.[57-59] The electronic self-consistent iteration and force convergence requirements were set to 10^{-4} eV and 0.02 eV, respectively.

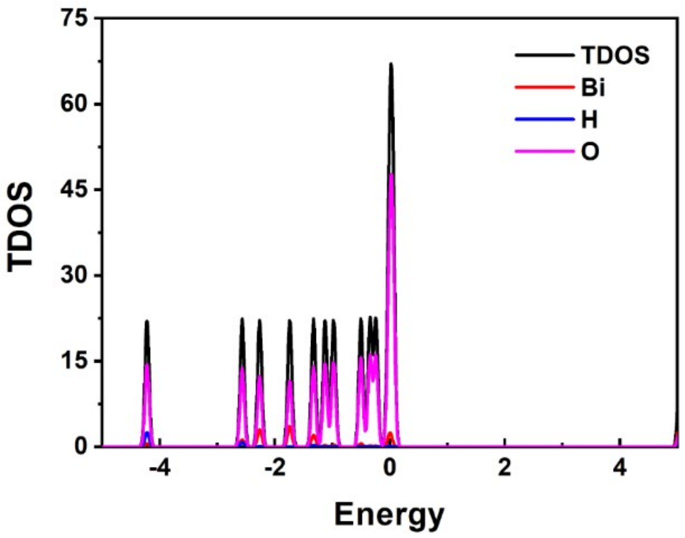

Fig. S20. The density of states of active Bi coordinative unsaturation with 4 oxygen atoms and one H_2O molecular bonded.

Reference

(1) Cao, C.; Ma, D.-D.; Jia, J.; Xu, Q.; Wu, X.-T.; Zhu, Q.-L. Divergent Paths, Same Goal: A Pair-Electrosynthesis Tactic for Cost-Efficient and Exclusive Formate Production by Metal-Organic-Framework-Derived 2D Electrocatalysts. *Adv. Mater.,* **2021**, *33*, 2008631.

(2) Wu, X.; Sun, J. W.; Liu, P. F.; Zhao, J. Y.; Liu, Y.; Guo, L.; Dai, S.; Yang, H. G.; Zhao, H. Molecularly dispersed cobalt phthalocyanine mediates selective and durable CO_2 reduction in a membrane flow cell. *Adv. Funct. Mater.,* **2022**, *32*, 2107301.

(3) Yi, J. D.; Xie, R.; Xie, Z. L.; Chai, G. L.; Liu, T. F.; Chen, R. P.; Huang, Y. B.; Cao, R. Highly Selective CO_2 Electroreduction to CH_4 by In Situ Generated Cu_2O Single-Type Sites on a Conductive MOF: Stabilizing Key Intermediates with Hydrogen Bonding. *Angew. Chem. Int. Ed.,* **2020**, *59*, 23641-23648.

(4) An, B.; Li, Z.; Song, Y.; Zhang, J.; Zeng, L.; Wang, C.; Lin, W. Cooperative copper centres in a metal-organic framework for selective conversion of CO_2 to ethanol. *Nature Catal.,* **2019**, *2*, 709-717.

(5) Wang, S.; Wahiduzzaman, M.; Davis, L.; Tissot, A.; Shepard, W.; Marrot, J.; Martineau-Corcos, C.; Hamdane, D.; Maurin, G.; Devautour-Vinot, S.; et al. A robust zirconium amino acid metal-organic framework for proton conduction. *Nat. Commun.,* **2018**, *9*, 4937.

(6) Aubrey, M. L.; Wiers, B. M.; Andrews, S. C.; Sakurai, T.; Reyes-Lillo, S. E.; Hamed, S. M.; Yu, C.-J.; Darago, L. E.; Mason, J. A.; Baeg, J.-O.; et al. Electron delocalization and charge mobility as a function of reduction in a metal-organic framework. *Nat. Mater.,* **2018**, *17*, 625-632.

(7) Sun, L.; Park, S. S.; Sheberla, D.; Dincă, M. Measuring and Reporting Electrical Conductivity in Metal-Organic Frameworks: Cd_2(TTFTB) as a Case Study. *J. Am. Chem. Soc.,* **2016**, *138*, 14772-14782.

(8) Inukai, M.; Horike, S.; Itakura, T.; Shinozaki, R.; Ogiwara, N.; Umeyama, D.; Nagarkar, S.; Nishiyama, Y.; Malon, M.; Hayashi, A.; et al. Encapsulating Mobile Proton Carriers into Structural Defects in Coordination Polymer Crystals: High Anhydrous Proton Conduction and Fuel Cell Application. *J. Am. Chem. Soc.,* **2016**, *138*, 8505-8511.

(9) Park, J.; Hinckley, A. C.; Huang, Z.; Chen, G.; Yakovenko, A. A.; Zou, X.; Bao, Z. High Thermopower in a Zn-Based 3D Semiconductive Metal-Organic Framework. *J. Am. Chem. Soc.,* **2020**, *142*, 20531-20535.

(10) Choi, J. Y.; Flood, J.; Stodolka, M.; Pham, H. T. B.; Park, J. From 2D to 3D: Postsynthetic Pillar Insertion in Electrically Conductive MOF. *ACS Nano,* **2022**, *16*, 3145-3151.

(11) Kung, C.-W.; Otake, K.; Buru, C. T.; Goswami, S.; Cui, Y.; Hupp, J. T.; Spokoyny, A. M.; Farha, O. K. Increased Electrical Conductivity in a Mesoporous Metal-Organic Framework Featuring Metallacarboranes Guests. *J. Am. Chem. Soc.,* **2018**, *140*, 3871-3875.

(12) Xie, L. S.; Sun, L.; Wan, R.; Park, S. S.; DeGayner, J. A.; Hendon, C. H.; Dincă, M. Tunable mixed-valence doping toward record electrical conductivity

in a three-dimensional metal-organic framework. *J. Am. Chem. Soc.,* **2018**, *140*, 7411-7414.

(13) Lian, Y.; Yang, W.; Zhang, C.; Sun, H.; Deng, Z.; Xu, W.; Song, L.; Ouyang, Z.; Wang, Z.; Guo, J. Unpaired 3d electrons on atomically dispersed cobalt centres in coordination polymers regulate both oxygen reduction reaction (ORR) activity and selectivity for use in zinc-air batteries. *Angew. Chem. Int. Ed.,* **2020**, *59*, 286-294.

(14) Huang, X.; Sheng, P.; Tu, Z.; Zhang, F.; Wang, J.; Geng, H.; Zou, Y.; Di, C.-a.; Yi, Y.; Sun, Y. A two-dimensional π–d conjugated coordination polymer with extremely high electrical conductivity and ambipolar transport behaviour. *Nat. Commun.,* **2015**, *6*, 1-8.

(15) Cao, Z.; Derrick, J. S.; Xu, J.; Gao, R.; Gong, M.; Nichols, E. M.; Smith, P. T.; Liu, X.; Wen, X.; Copéret, C.; et al. Chelating N-Heterocyclic Carbene Ligands Enable Tuning of Electrocatalytic CO_2 Reduction to Formate and Carbon Monoxide: Surface Organometallic Chemistry. *Angew. Chem. Int. Ed.,* **2018**, *57*, 4981-4985.

(16) Liu, S.-Q.; Shahini, E.; Gao, M.-R.; Gong, L.; Sui, P.-F.; Tang, T.; Zeng, H.; Luo, J.-L. Bi_2O_3 Nanosheets Grown on Carbon Nanofiber with Inherent Hydrophobicity for High-Performance CO_2 Electroreduction in a Wide Potential Window. *ACS Nano,* **2021**, *15*, 17757-17768.

(17) Liu, S.; Lu, X. F.; Xiao, J.; Wang, X.; Lou, X. W. Bi_2O_3 Nanosheets Grown on Multi-Channel Carbon Matrix to Catalyze Efficient CO_2 Electroreduction to HCOOH. *Angew. Chem. Int. Ed.,* **2019**, *58*, 13828-13833.

(18) Feng, X.; Zou, H.; Zheng, R.; Wei, W.; Wang, R.; Zou, W.; Lim, G.; Hong, J.; Duan, L.; Chen, H. Bi_2O_3/BiO_2 Nanoheterojunction for Highly Efficient Electrocatalytic CO_2 Reduction to Formate. *Nano Lett.,* **2022**, *22*, 1656-1664.

(19) Kong, L.; Zhong, M.; Shuang, W.; Xu, Y.; Bu, X.-H. Electrochemically active sites inside crystalline porous materials for energy storage and conversion. *Chem. Soc. Rev.,* **2020**, *49*, 2378-2407.

(20) Wang, L.; Liu, P.; Xu, Y.; Zhao, Y.; Xue, N.; Guo, X.; Peng, L.; Zhu, Y.; Ding, M.; Wang, Q.; et al. Enhanced catalytic activity and stability of bismuth nanosheets decorated by 3-aminopropyltriethoxysilane for efficient electrochemical reduction of CO_2. *Appl. Catal. B,* **2021**, *298*, 120602.

(21) Cao, C.; Ma, D. D.; Jia, J.; Xu, Q.; Wu, X. T.; Zhu, Q. L. Divergent Paths, Same Goal: A Pair-Electrosynthesis Tactic for Cost-Efficient and Exclusive Formate Production by Metal-Organic-Framework-Derived 2D Electrocatalysts. *Adv. Mater.,* **2021**, *33*, 2008631.

(22) Wang, Y.; Huang, Z.; Lei, Y.; Wu, J.; Bai, Y.; Zhao, X.; Liu, M.; Zhan, L.; Tang, S.; Zhang, X.; et al. Bismuth with abundant defects for electrocatalytic CO_2 reduction and Zn-CO_2 batteries. *Chem. Commun.,* **2022**, *58*, 3621-3624,

(23) Lee, J.; Liu, H.; Chen, Y.; Li, W. Bismuth Nanosheets Derived by In Situ Morphology Transformation of Bismuth Oxides for Selective Electrochemical CO_2 Reduction to Formate. *ACS Appl. Mater. Interfaces,* **2022**, *14*, 14210-14217.

(24) Cao, C.; Ma, D.-D.; Gu, J.-F.; Xie, X.; Zeng, G.; Li, X.; Han, S.-G.; Zhu, Q.-L.; Wu, X.-T.; Xu, Q. Metal-Organic Layers Leading to Atomically Thin Bismuthene for Efficient Carbon Dioxide Electroreduction to Liquid Fuel. *Angew. Chem. Int. Ed.,* **2020**, *59*, 15014-15020.
(25) Li, J.; Wang, C.; Wang, D.; Yang, C.; Cui, X.; Gao, X. J.; Zhang, Z. A hexacoordinated Bi^{3+}-based ellagate MOF with acid/base resistance boosting carbon dioxide electroreduction to formate. *J. Mater. Chem. A*, **2022**, *10*, 20018-20023.
(26) Li, F.; Gu, G. H.; Choi, C.; Kolla, P.; Hong, S.; Wu, T.-S.; Soo, Y.-L.; Masa, J.; Mukerjee, S.; Jung, Y.; et al. Highly stable two-dimensional bismuth metal-organic frameworks for efficient electrochemical reduction of CO_2. *Appl. Catal. B,* **2020**, *277*, 119241.
(27) Zheng, H.; Wu, G.; Gao, G.; Wang, X. The bismuth architecture assembled by nanotubes used as highly efficient electrocatalyst for CO_2 reduction to formate. *Chem. Eng. J.,* **2021**, *421*, 129606.
(28) Wu, Z.; Wu, H.; Cai, W.; Wen, Z.; Jia, B.; Wang, L.; Jin, W.; Ma, T. Engineering Bismuth-Tin Interface in Bimetallic Aerogel with a 3D Porous Structure for Highly Selective Electrocatalytic CO_2 Reduction to HCOOH. *Angew. Chem. Int. Ed.,* **2021**, *133*, 12662-12667.
(29) Yao, D.; Tang, C.; Vasileff, A.; Zhi, X.; Jiao, Y.; Qiao, S. Z. The Controllable Reconstruction of Bi-MOFs for Electrochemical CO_2 Reduction through Electrolyte and Potential Mediation. *Angew. Chem. Int. Ed.,* **2021**, *60*, 18178-18184.
(30) Zhang, M.; Wei, W.; Zhou, S.; Ma, D.-D.; Cao, A.; Wu, X.-T.; Zhu, Q.-L. Engineering a conductive network of atomically thin bismuthene with rich defects enables CO_2 reduction to formate with industry-compatible current densities and stability. *Energy Environ. Sci.,* **2021**, *14*, 4998-5008.
(31) Lamagni, P.; Miola, M.; Catalano, J.; Hvid, M. S.; Mamakhel, M. A. H.; Christensen, M.; Madsen, M. R.; Jeppesen, H. S.; Hu, X.-M.; Daasbjerg, K.; et al. Restructuring Metal-Organic Frameworks to Nanoscale Bismuth Electrocatalysts for Highly Active and Selective CO_2 Reduction to Formate. *Adv. Funct. Mater.,* **2020**, *30*, 1910408.
(32) Zhang, W.; Yang, S.; Jiang, M.; Hu, Y.; Hu, C.; Zhang, X.; Jin, Z. Nanocapillarity and Nanoconfinement Effects of Pipet-like Bismuth@Carbon Nanotubes for Highly Efficient Electrocatalytic CO_2 Reduction. *Nano Lett.,* **2021**, *21*, 2650-2657.
(33) Zhang, X.; Sun, X.; Guo, S.-X.; Bond, A. M.; Zhang, J. Formation of lattice-dislocated bismuth nanowires on copper foam for enhanced electrocatalytic CO_2 reduction at low overpotential. *Energy Environ. Sci.,* **2019**, *12*, 1334-1340.
(34) Gong, Q.; Ding, P.; Xu, M.; Zhu, X.; Wang, M.; Deng, J.; Ma, Q.; Han, N.; Zhu, Y.; Lu, J. Structural defects on converted bismuth oxide nanotubes enable highly active electrocatalysis of carbon dioxide reduction. *Nat. Commun.,* **2019**, *10*, 1-10.
(35) Zhu, J.; Fan, J.; Cheng, T.; Cao, M.; Sun, Z.; Zhou, R.; Huang, L.; Wang, D.;

Li, Y.; Wu, Y. Bilayer nanosheets of unusual stoichiometric bismuth oxychloride for potassium ion storage and CO_2 reduction. *Nano Energy,* **2020**, *75*, 104939.

(36) Wang, Z.; Zhou, Y.; Xia, C.; Guo, W.; You, B.; Xia, B. Y. Efficient Electroconversion of Carbon Dioxide to Formate by a Reconstructed Amino-Functionalized Indium-Organic Framework Electrocatalyst. *Angew. Chem. Int. Ed.,* **2021**, *60*, 19107-19112.

(37) Ma, W.; Xie, S.; Zhang, X.-G.; Sun, F.; Kang, J.; Jiang, Z.; Zhang, Q.; Wu, D.-Y.; Wang, Y. Promoting electrocatalytic CO_2 reduction to formate via sulfur-boosting water activation on indium surfaces. *Nat. Commun.,* **2019**, *10*, 892.

(38) Zhou, S.; Kong, X.; Zheng, B.; Huo, F.; Strømme, M.; Xu, C. Cellulose Nanofiber @ Conductive Metal-Organic Frameworks for High-Performance Flexible Supercapacitors. *ACS Nano,* **2019**, *13*, 9578-9586.

(39) Luo, Y.-h.; Pan, Q.-l.; Wei, H.-x.; Huang, Y.-d.; Tang, L.-b.; Wang, Z.-y.; He, Z.-j.; Yan, C.; Mao, J.; Dai, K.-h.; et al. Towards Ni-rich layered oxides cathodes with low Li/Ni intermixing by mild molten-salt ion exchange for lithium-ion batteries. *Nano Energy,* **2022**, *102*, 107626.

(40) Zhao, J.; Cheng, H.; Zhang, Z.; Liu, Y.; Song, J.; Liu, T.; He, Y.; Meng, A.; Sun, C.; Hu, M.; et al. The Semicoherent Interface and Vacancy Engineering for Constructing $Ni(Co)Se_2$@$Co(Ni)Se_2$ Heterojunction as Ultrahigh-Rate Battery-Type Supercapacitor Cathode. *Adv. Funct. Mater.,* **2022**, *32*, 2202063.

(41) Duan, H.; Zhao, Z.; Lu, J.; Hu, W.; Zhang, Y.; Li, S.; Zhang, M.; Zhu, R.; Pang, H. When Conductive MOFs Meet MnO2: High Electrochemical Energy Storage Performance in an Aqueous Asymmetric Supercapacitor. *ACS Appl. Mater. Interfaces,* **2021**, *13*, 33083-33090.

(42) Tian, J.; Cao, C.; Ma, D.-D.; Han, S.-G.; He, Y.; Wu, X.-T.; Zhu, Q.-L. Killing Two Birds with One Stone: Selective Oxidation of Small Organic Molecule as Anodic Reaction to Boost CO_2 Electrolysis. *Small Structures,* **2022**, *3*, 2100134.

(43) Mesbah, A.; Rabu, P.; Sibille, R. S. b. Lebegue, T. Mazet, B. Malaman and M. Francois. *Inorg. Chem.,* **2014**, *53*, 872-881.

(44) Ai, L.; Zhang, C.; Li, L.; Jiang, J. Iron terephthalate metal-organic framework: revealing the effective activation of hydrogen peroxide for the degradation of organic dye under visible light irradiation. *Appl. Catal. B,* **2014**, *148*, 191-200.

(45) Li, J.; Wei, R.; Wang, X.; Zuo, Y.; Han, X.; Arbiol, J.; Llorca, J.; Yang, Y.; Cabot, A.; Cui, C. Selective methanol-to-formate electrocatalytic conversion on branched nickel carbide. *Angew. Chem. Int. Ed.,* **2020**, *132*, 21012-21016.

(46) Wang, L.; Zhu, Y.; Wen, Y.; Li, S.; Cui, C.; Ni, F.; Liu, Y.; Lin, H.; Li, Y.; Peng, H. Regulating the local charge distribution of Ni active sites for the urea oxidation reaction. *Angew. Chem. Int. Ed.,* **2021**, *133*, 10671-10676.

(47) Qi, Y.; Zhang, Y.; Yang, L.; Zhao, Y.; Zhu, Y.; Jiang, H.; Li, C. Insights into the activity of nickel boride/nickel heterostructures for efficient methanol electrooxidation. *Nat. Commun.,* **2022**, *13*, 1-11.

(48) Zhang, N.; Feng, X.; Rao, D.; Deng, X.; Cai, L.; Qiu, B.; Long, R.; Xiong, Y.; Lu, Y.; Chai, Y. Lattice oxygen activation enabled by high-valence metal sites

for enhanced water oxidation. *Nat. Commun.,* **2020**, *11*, 1-11.

(49) Haselmann, G. M.; Baumgartner, B.; Wang, J.; Wieland, K.; Gupta, T.; Herzig, C.; Limbeck, A.; Lendl, B.; Eder, D. In situ Pt photodeposition and methanol photooxidation on Pt/TiO_2: Pt-loading-dependent photocatalytic reaction pathways studied by liquid-phase infrared spectroscopy. *ACS Catal.,* **2020**, *10*, 2964-2977.

(50) Zalineeva, A.; Baranton, S.; Coutanceau, C. How do Bi-modified palladium nanoparticles work towards glycerol electrooxidation? An in situ FTIR study. *Electrochim. Acta,* **2015**, *176*, 705-717.

(51) Zhu, J.; Xia, L.; Yu, R.; Lu, R.; Li, J.; He, R.; Wu, Y.; Zhang, W.; Hong, X.; Chen, W. Ultrahigh stable methanol oxidation enabled by a high hydroxyl concentration on Pt clusters/MXene interfaces. *J. Am. Chem. Soc.,* **2022**, *144*, 15529-15538.

(52) Li, J.; Wei, R.; Wang, X.; Zuo, Y.; Han, X.; Arbiol, J.; Llorca, J.; Yang, Y.; Cabot, A.; Cui, C. Selective Methanol-to-Formate Electrocatalytic Conversion on Branched Nickel Carbide. *Angew. Chem. Int. Ed.,* **2020**, *59*, 20826-20830.

(53) Kresse, G.; Furthmüller, J. Efficiency of ab-initio total energy calculations for metals and semiconductors using a plane-wave basis set. *Comp. Mater. Sci.,* **1996**, *6*, 15-50.

(54) Kresse, G.; Furthmüller, J. Efficient iterative schemes for ab initio total-energy calculations using a plane-wave basis set. *Phys. Rev. B,* **1996**, *54*, 11169-11186.

(55) Perdew, J. P.; Burke, K.; Ernzerhof, M. Generalized Gradient Approximation Made Simple. *Phys. Rev. Lett.,* **1996**, *77*, 3865-3868.

(56) Blöchl, P. E. Projector augmented-wave method. *Phys. Rev. B,* **1994**, *50*, 17953-17979.

(57) Press, W.; Flannery, B.; Teukolsky, S.; Vetterling, W. Numerical Recipes: the Art of Computer Programming. New York: Cambridge University Press: 1986.

(58) Yang, W.; Li, J.; Cui, X.; Yang, C.; Liu, Y.; Zeng, X.; Zhang, Z.; Zhang, Q. Fine-tuning inverse metal-support interaction boosts electrochemical transformation of methanol into formaldehyde based on density functional theory. *Chin. Chem. Lett.,* **2021**, *32*, 2489-2494.

(59) Yang, W.; Zhu, Y.; Li, J.; Chen, Z.; Nosheen, F.; Zhang, Q.; Zhang, Z. Understanding the dehydrogenation mechanism over iron nanoparticles catalysts based on density functional theory. *Chin. Chem. Lett.,* **2021**, *32*, 286-290.
